# Enzymatic, cell-based, and *in silico* evaluation of di-substituted aminomethyl-1,2,3-triazole–cinamamide hybrids as mushroom tyrosinase inhibitors

**DOI:** 10.1039/d5ra04315h

**Published:** 2025-10-13

**Authors:** Navid Dastyafteh, Mohammad Hosein Sayahi, Mohammad Hossein Morshedsolouk, Sajedeh Safapoor, Mohammad Mahdavi, Mina Saeedi, Haleh Hamedifar, Nima Sepehri, Aida Iraji

**Affiliations:** a Stem Cells Technology Research Center, Shiraz University of Medical Sciences Shiraz Iran aida.iraji@gmail.com iraji@sums.ac.ir; b Department of Chemistry, Payame Noor University Tehran Iran; c Endocrinology and Metabolism Research Center, Endocrinology and Metabolism Clinical Sciences Institute, Tehran University of Medical Sciences Tehran Iran; d Persian Medicine and Pharmacy Research Center, Tehran University of Medical Sciences Tehran Iran; e Medicinal Plants Research Center, Faculty of Pharmacy, Tehran University of Medical Sciences Tehran Iran; f CinnaGen Medical Biotechnology Research Center, Alborz University of Medical Sciences Karaj 1461965381 Iran N.sepehri@nanoalvand.com; g CinnaGen Research and Production Co. Alborz 3164819712 Iran; h Research Center for Traditional Medicine and History of Medicine, Department of Persian Medicine, School of Medicine, Shiraz University of Medical Sciences Shiraz Iran

## Abstract

A series of novel aryl-substituted aminomethyl 1,2,3-triazole–cinamamide hybrids (9a–q) were synthesized and tested as tyrosinase inhibitors using enzymatic, *in silico*, and cell-based assays. A multi-step synthetic procedure, involving coupling *N*-(prop-2-yn-1-yl)cinnamamide intermediates with various azido-functionalized anilide derivatives and click chemistry, gives the final 1,2,3-triazole-linked hybrids in good yields. SAR studies pointed out that compound 9i (R^1^ = 4-Cl, R^2^ = 4-Br) was the best tyrosinase inhibitor and was found to be a promising antioxidant in DPPH radical scavenging (51.82% at 200 µM). Kinetic studies on the enzyme reveal that the inhibition type is competitive with a *K*_i_ value of 34.36 µM. Further molecular docking and molecular dynamics simulations supported the strong binding interaction of 9i in the tyrosinase active site *via* π–π stacking interactions with the His residues and hydrogen bonding with important catalytic residues. Consistent with these findings, *in vitro* studies showed that 9i had low cytotoxicity to B16F10 melanoma cells at concentrations ≤100 µM but was able to reduce intracellular melanin content from 92 to 62 µg mL^−1^. Therefore, compound 9i represents a potent tyrosinase inhibitor with antioxidant and anti-melanogenic properties, which might further assist in the development of anti-melanoma agents.

## Abstract 2

A series of novel aryl-substituted aminomethyl 1,2,3-triazole–cinamamide hybrids (9a–q) were synthesized and tested as tyrosinase inhibitors using enzymatic, *in silico*, and cell-based assays.

## Graphical abstract

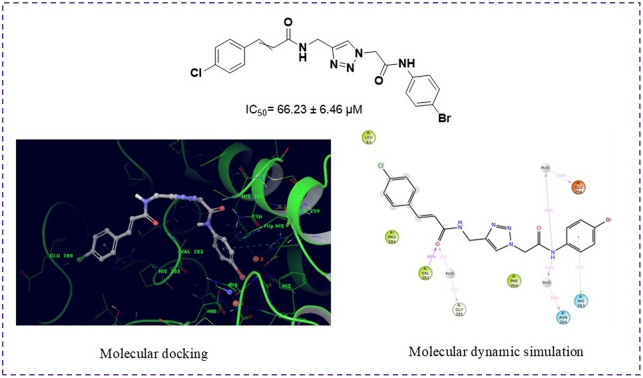

## 1. Introduction

Tyrosinase (polyphenol oxidase, EC 1.14.18.1) is a multifunctional copper-containing enzyme found in various organisms, from bacteria to plants to animals. It is a very important enzyme in melanin biosynthesis since it hydroxylates l-tyrosine to l-DOPA (3,4-dihydroxyphenylalanine) and then oxidizes l-DOPA to dopaquinone, a principal precursor in melanin synthesis.^1^ The enzyme is crucial for skin pigment formation and causes enzymatic browning in plants and cuticle formation in insects. Overactivation in tyrosinase activity leads to hyperpigmentation disorders, such as melasma, age spots, and post-inflammatory hyperpigmentation, which seriously mar the cosmetic appeal of patients and resultant psychological trauma.^2,3^ While atypical melanin production is associated with the exacerbation of melanoma, tyrosinase inhibitors could aid in treating skin cancers. Furthermore, more recent studies point toward possible roles tyrosinase may play in neurodegenerative disorders such as Parkinson's, where dopamine metabolism is impaired.^4^

In the food industry, tyrosinase-mediated enzymatic browning occurs, leading to deteriorating quality in fruits, vegetables, and seafood.^5^ Browning destroys visual appeal and, by extension, depletes nutritional potential through the degradation of active compounds and vitamins. Traditional inhibitors such as sulfites and ascorbic acid have been limited by regulatory restrictions and inconsistent efficiency.^6^ Thus, a novel tyrosinase inhibitor, whether natural or synthetic, should be identified with a better safety profile and improved stability for food preservation and extended shelf life.

**Fig. 1:**
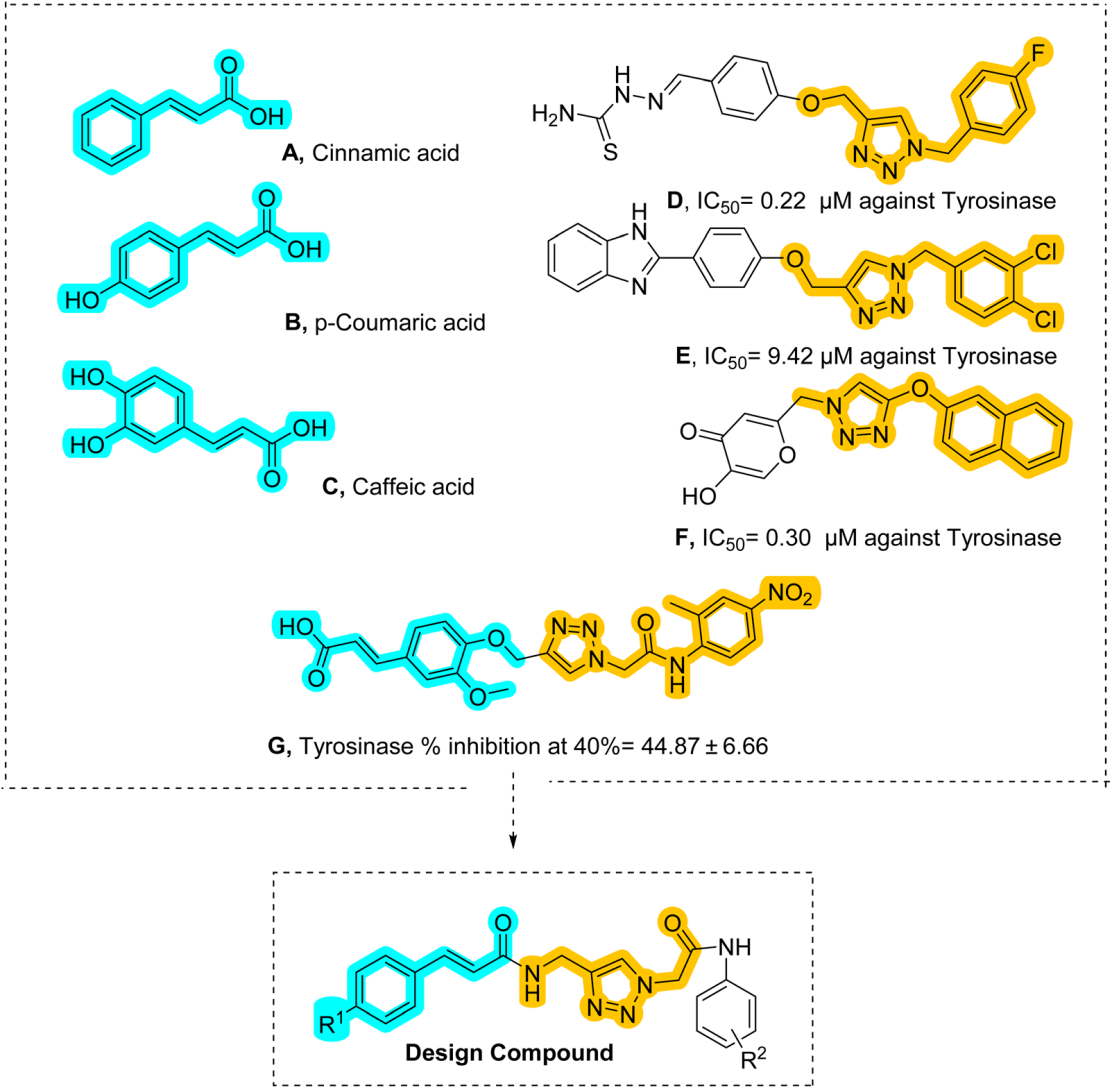
Designing.

Cinnamic acid (A, Fig. 1) and its derivatives are regarded as potent tyrosinase inhibitors, due to their resemblance to tyrosine, the enzyme's natural substrate. The presence of this α,β-unsaturated carbonyl moiety renders these compounds competitive inhibitors, targeting the enzyme's active site.^7^ Substitution on the phenyl ring with hydroxyl or methoxy groups has been reported to improve inhibitory activity greatly . *p*-Coumaric acid (B, Fig. 1) was found to be more potent than cinnamic acid, probably due to the extra hydrogen bonding with the enzyme.^8^ Likewise, ferulic acid has been reported to exert a very strong anti-melanogenic effect on B16F10 melanoma cells, suggesting it could have applications in cosmetics and therapeutics.^9^ Caffeic acid (3,4-dihydroxycinnamic acid, C, Fig. 1), being more active than mono-substituted derivatives, presumably chelates copper better at the enzyme active site.^10^ Nonetheless, some cinnamic acid derivatives are limited in use due to their poor stability under UV light; thus, hybrid molecules with better properties are being investigated.

Amino 1,2,3-triazoles (D–F, Fig. 1) have always been of interest as tyrosinase inhibitors. Different reports on this particular class of compounds have pointed out their ability to form coordination complexes with copper ions at the active site of the enzyme.^11,12^ In recent decades, researchers have put much effort into modifying the 1,2,3-triazole scaffold to achieve selective and potent action against tyrosinase.^13–15^

Hybrid molecules with phenolic moieties conjugated to 1,2,3-triazoles showed better inhibition. Drug design efforts have recently investigated hybrid molecules combining a cinnamic acid and 1,2,3-triazole pharmacophore (G, Fig. 1) as a tyrosinase inhibitor. In the enzymatic assays, the most potent compound showed around 45% inhibition at 40 µM. SAR studies on this hybrid halogen, because of their electron-withdrawing nature, enhance inhibition of tyrosinase by stabilizing the enzyme–inhibitor complex, and electron donors at the ortho position tend to enhance inhibition as well.^16^

As a result of the current study, cinnamic derivatives mimicking the natural substrate, l-tyrosine, and the 1,2,3-triazole moiety, which enhances binding affinity with the enzyme active site, were selected as the primary backbone. The design strategy involves optimizing the cinnamic ring (R^1^) with different groups as well as modifying the 1,2,3-triazole (R^2^) with flexible linkers. *In vitro* assays, cell toxicity, and melanin determination in B16F10 cells, along with molecular docking and molecular dynamics simulations, were conducted to predict key interactions.

## 2. Results and discussion

### 2.1 Chemistry

**Scheme 1:**
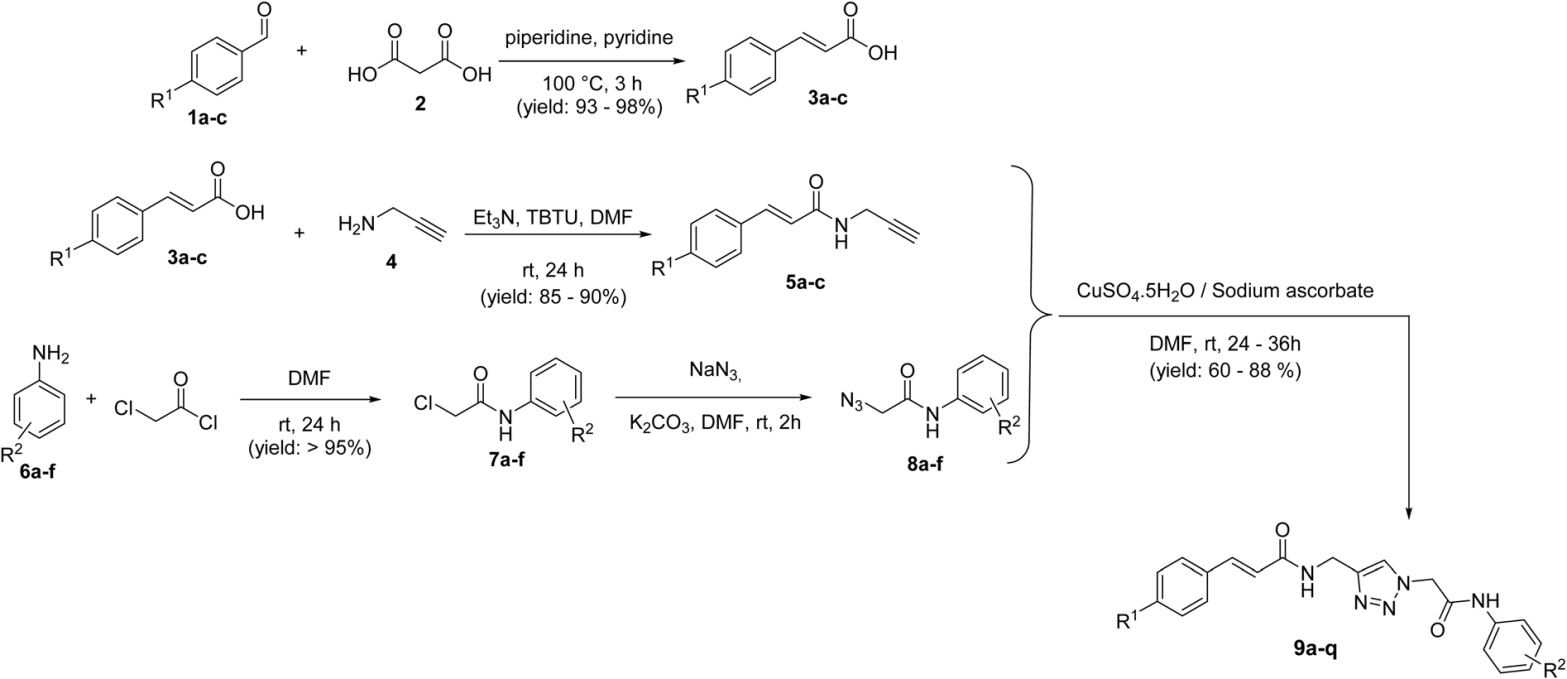
Synthesis of 9a–q.

A mixture containing benzaldehyde derivatives 1a–c, malonic acid, pyridine, and piperidine as a catalyst was stirred at 100 °C for 3 hours. Afterward, water was added, and the mixture was neutralized with concentrated HCl, yielding cinnamic acid derivatives 3a–c. To a solution of cinnamic acid derivatives 3a–c and propargylamine 4 in DMF, TBTU and Et_3_N were added. The mixture was stirred at room temperature for 24 hours. Upon completion of the reaction, water was added, and the resulting precipitate was filtered to obtain pure *N*-(prop-2-yn-1-yl)cinnamamide derivatives 5a–c. Aniline derivatives 6a–f were suspended in DMF and cooled to 0 °C. Chloroacetyl chloride was then added, and the reaction mixture was stirred at room temperature for 24 hours. After completion, the mixture was diluted with water, poured over crushed ice, and the resulting precipitate was filtered. The residue was washed with water to obtain 2-chloro-*N*-substituted acetamide derivatives 7a–f. Next, 7a–f, K _2_CO_3,_ and NaN_3_ were dissolved in DMF and stirred at room temperature for 1 hour. According to Sharpless and co-workers' report for regioselective synthesis, 1,4-disubstituted 1,2,3-triazoles were obtained by the addition of copper sulfate pentahydrate (CuSO_4_·5H_2_O), sodium ascorbate, and *N*-(prop-2-yn-1-yl)cinnamamide derivatives 5a–c.^17^ Upon completion of the reaction, copper ions were removed using an EDTA solution. The resulting precipitate was filtered, washed several times with water, and crystallized from ethyl acetate to yield pure *N*-((1*H*-1,2,3-triazol-4-yl)methyl)cinnamamide-*N*-substituted acetamides 9a–q (Scheme 1).

The structures of the synthesized compounds were elucidated using NMR and IR spectroscopy. The purity of compound 9i was confirmed using HPLC, and compound 9q was further characterized using high-resolution mass spectrometry (HRMS), which provided accurate mass data supporting its structural assignment.

### 2.2 Structure–activity relationship (SAR) analysis of 9a–p against tyrosinase

The SAR analysis of these compounds reveals clear trends that depend on the R^1^ and R^2^ substitutions. The parent compound 9a, where R^2^ is hydrogen, is almost inactive (IC_50_ > 200 µM). The incorporation of 4-F on R^2^ (9b) enhances the potency (IC_50_ = 158.53 µM), whereas 4-Cl (9c) has moderate potency (IC_50_ = 190.76 µM); the best activity within the set returned to 4-Br (9d) with IC_50_ = 142.56 µM. The 4-ethyl group (9e) offers the same moderate improvement (IC_50_ = 178.23 µM), as electron-withdrawing at R^2^ enhances activity compared to the unsubstituted analog 9a, but is not enough to achieve strong inhibition.

The substitution of R^1^ with 4-Cl (9f–k) is associated with an overall enhancement of the activity relative to the R^1^=H set. The parent R^2^-unsubstituted compound (9f) shows intermediate potency (IC_50_ = 153.03 µM), and the introduction of 4-F to R^2^ (9g) enhances the activity (IC_50_ = 138.37 µM). Significantly, the introduction of 4-Cl at both R^1^ and R^2^ (9h) leads to the loss of potency (IC_50_ > 200 µM), indicating steric hindrance and/or electronic repulsion. The optimal compound in the set is 9i (*R*^1^ = 4-Cl, *R*^2^ = 4-Br), which is the most active (IC_50_ = 66.23 µM), followed by 9j (*R*^2^ = 4-ethyl, IC_50_ = 96.32 µM). This indicates that bulkier or more polarizable R^2^ substituents, such as 4-Br, greatly increase the activity in the set. Compound 9k bearing a 4-MeO substituent showed markedly reduced potency, with IC_50_ > 200 µM, indicating that heteroatom-based electron-donating substitution is not beneficial.

In the case of the *R*^1^ = 4-MeO derivatives (9l–q), the trend is as expected with moderate activity across various R^2^ substituents. The parent compound (9l) showed an IC_50_ of 165.57 µM with improvements seen in 4-F (9m, IC_50_ = 158.56 µM), 4-Cl (9n, IC_50_ = 148.01 µM), and 4-Br (9o, IC_50_ = 139.48 µM). The 4-ethyl substituent (9p) affords an IC_50_ of 153.15 µM.

Compound 9q bearing MeO substitution at R^1^ and R^2^ shows IC_50_ = 182.86 ± 5.54, so that such bulk electron donating does not improve the potency *vs.*9i.

Overall, SAR trends indicate that the type of substitution at R^2^ has a significant impact on activity. The most active compound, 9i (R^1^: 4-Cl, R^2^: 4-Br), highlights the critical role of halogen-mediated interactions in increasing potency. The introduction of electron-withdrawing groups at R^2^, such as 4-F and 4-Cl, results in moderate potency. In contrast, the 4-Br substituent leads to increased activity, likely due to its bulk and polarizability. Furthermore, evaluations of the nature of the substitution at R^1^ indicated that chlorine was superior to unsubstitution and MeO substitution at R^1^ (Table 1).

**Table 1:** Tyrosinase inhibitory activities of 9a–q^a^

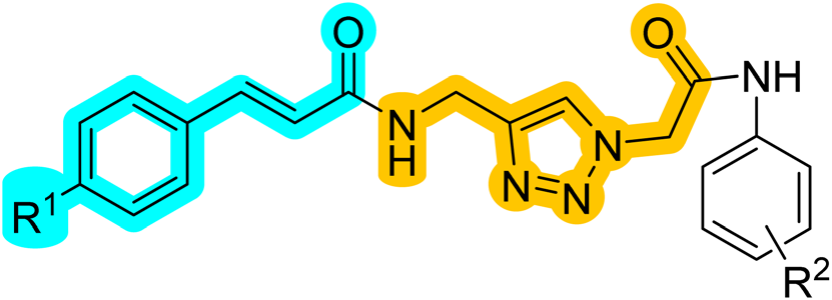			
Compound	R^1^	R^2^	IC_50_ µM
9a	H	H	> 200
9b	H	4-F	158.53 ± 5.16
9c	H	4-Cl	190.76 ± 0.31
9d	H	4-Br	142.56 ± 0.46
9e	H	4-Ethyl	178.23 ± 11.89
9f	4-Cl	H	153.03 ± 7.72
9g	4-Cl	4-F	138.37 ± 13.49
9h	4-Cl	4-Cl	> 200
9i	4-Cl	4-Br	66.23 ± 6.46
9j	4-Cl	4-Ethyl	96.32 ± 4.55
9k	4-Cl	4-MeO	> 200
9l	4-MeO	H	165.57 ± 11.04
9m	4-MeO	4-F	158.56 ± 6.71
9n	4-MeO	4-Cl	148.01 ± 7.71
9o	4-MeO	4-Br	139.48 ± 0.68
9p	4-MeO	4-Ethyl	153.15 ± 18.65
9q	4-MeO	4-MeO	182.86 ± 5.54

a Kojic acid as positive control showed IC_50_ = 27.56 ± 1.27 µM.

### 2.3 Antioxidant activity potential

The synthesized compounds are tested for their antioxidant activity *via* the DPPH radical scavenging at 200 µM. The effect of substituents at R^1^ or R^2^ positions on the % inhibition of DPPH radicals was studied to understand SAR. Quercetin, as a positive control, showed IC_50_ = 18.56 ± 2.19 µM.

Compound 9a (R^1^: H, R^2^: H) exhibited the lowest inhibition (23.64%). The introduction of fluorine into the R^2^ position (9b; R^1^: H, R^2^: 4-F) caused a notable increase in the antioxidant capacity (64.18%). Substitution with bromine at R^2^ (9d; R^1^: H, R^2^: 4-Br) showed a moderate result (41.09%), while chlorine at R^2^ (9c, R^1^: H, R^2^: 4-Cl) showed a slightly lower effect (36.91%).

Among compounds with R^1^ = 4-Cl, the presence of 4-Br at R^2^ (9i) and 4-ethyl at R^2^ (9j ) resulted in increased activity. Compound 9j (R^1^: 4-Cl, R^2^: 4-ethyl) exhibited the highest inhibition (82.36%), indicating that the presence of both a chlorine at R^1^ and an ethyl group at R^2^ significantly enhances activity. 9k bearing 4-MeO also exhibited good antioxidant activity with 55.28 ± 0.16% inhibition. A methoxy (–OMe) substitution at R^1^ (9l–q) enhanced antioxidant activity compared to hydrogen (9a). Among these, 9n (R^1^: 4-MeO, R^2^: 4-Cl) exhibited the highest activity (68.64%). The combination of 4-MeO at R^1^ with halogens at R^2^ (9m: R^2^: 4-F, 60.18%; 9n: R^2^: 4-Cl, 68.64%; 9o: R^2^: 4-Br, 57.09%) improved antioxidant properties. The presence of two methoxy groups in compound 9q was also beneficial, resulting in 61.51% inhibition.

The overall trend is that an electron-withdrawing group, especially halogens such as fluorine and bromine, at R^2^ enhances radical scavenging, and at R^2^, bulky alkyl groups such as ethyl considerably enhance the activity. However, the best free radical scavenging activity was accounted for compound 9j (4-Cl, 4-ethyl) with 82.36% free radical scavenging. Also, the methoxy substitution at R^1^ facilitates improved activity, particularly with halogens at R^2^, whereas the lowest activity was recorded with those lacking substituents or having excessive halogenation, such as 9h (R^1^: 4-Cl, R^2^: 4-Cl) (Table 2).

**Table 2:** The antioxidant potential of 9a–q^a^

Compound	R^1^	R^2^	% Inhibition at 200 µM
9a	H	H	23.64 ± 4.63
9b	H	4-F	64.18 ± 2.31
9c	H	4-Cl	36.91 ± 0.51
9d	H	4-Br	41.09 ± 4.11
9e	H	4-Ethyl	47.36 ± 1.93
9f	4-Cl	H	46.18 ± 1.54
9g	4-Cl	4-F	45.73 ± 3.47
9h	4-Cl	4-Cl	22.82 ± 0.90
9i	4-Cl	4-Br	51.82 ± 2.31
9j	4-Cl	4-Ethyl	82.36 ± 5.91
9k	4-Cl	4-MeO	55.28 ± 0.16
9l	4-MeO	H	43.91 ± 4.50
9m	4-MeO	4-F	60.18 ± 6.94
9n	4-MeO	4-Cl	68.64 ± 7.33
9o	4-MeO	4-Br	57.09 ± 6.17
9p	4-MeO	4-Ethyl	40.73 ± 5.91
9q	4-MeO	4-MeO	61.51 ± 3.74

a Quercetin as positive control; IC_50_ = 18.56 ± 2.19 µM.

### 2.4 Enzyme kinetic studies

**Fig. 2:**
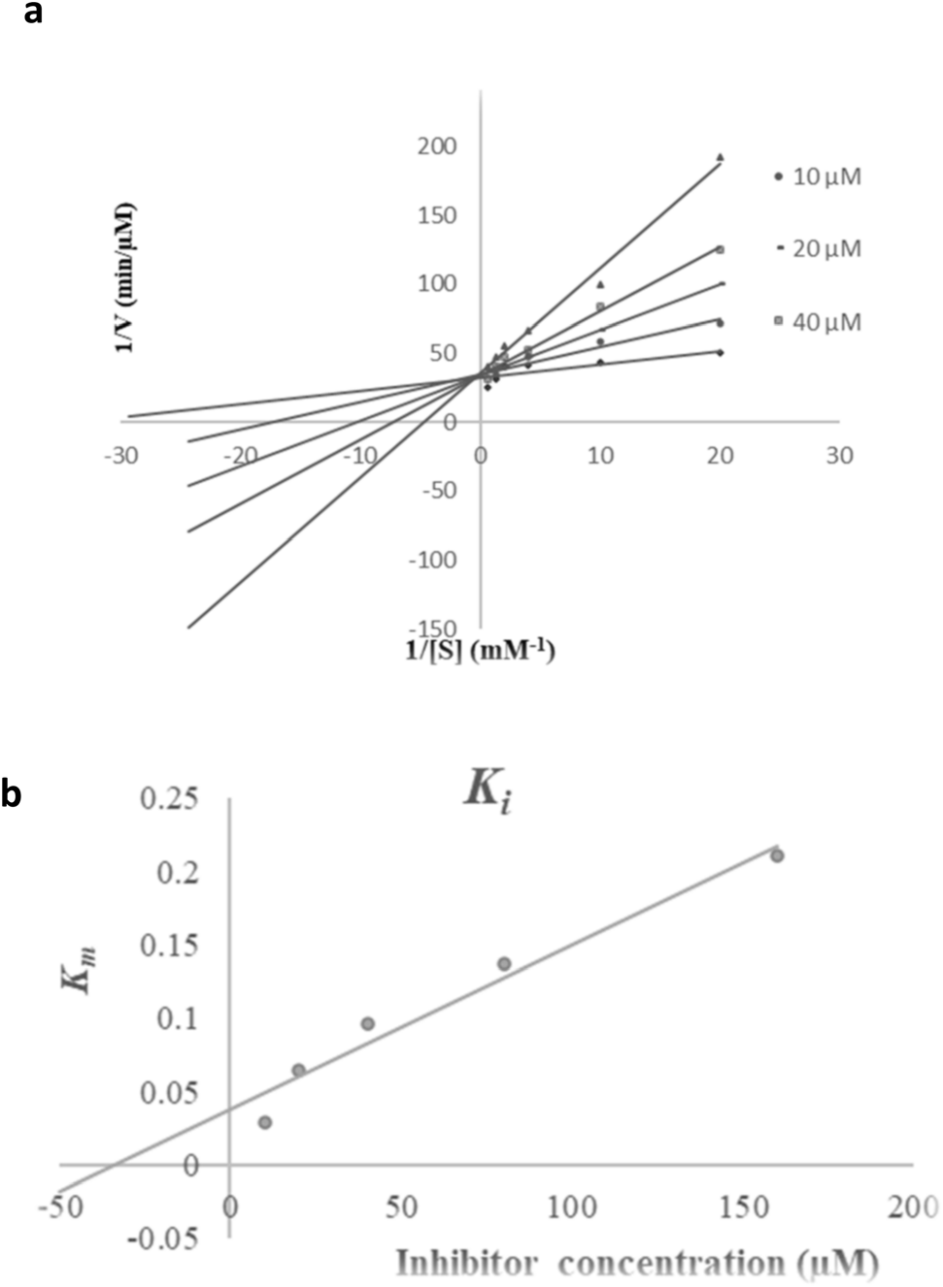
Kinetics of tyrosinase inhibition by 9i. (a) The Lineweaver–Burk plot at different concentrations of 9i; (b) the secondary plot between *K*_m_ and various concentrations of 9i.

To gain insight into the mechanism of action of 9i as the most potent tyrosinase inhibitor, kinetic measurements were performed. According to Fig. 2a, the Lineweaver–Burk plot showed that *K*_m_ gradually increased and *V*_max_ remained unchanged with increasing inhibitor concentration, indicating competitive inhibition. The results show 9i interacts with the active site on the enzyme and competes with the substrate for binding to the active site. Furthermore, the plot of the *K*_m_*versus* different concentrations of inhibitor gave an estimate of the inhibition constant, *K*_i_ of 34.36 µM (Fig. 2b).

### 2.5 Molecular docking and molecular dynamics study

To provide docking studies, an initial validation was performed. As a result, tropolone, the co-crystallized ligand, which is an established tyrosinase inhibitor, was docked into the tyrosinase active site. The Root Mean Square Deviation (RMSD) value between the docked pose and the crystallographic conformation was less than 2.0 Å, which confirms that the docking protocol is valid.

**Fig. 3:**
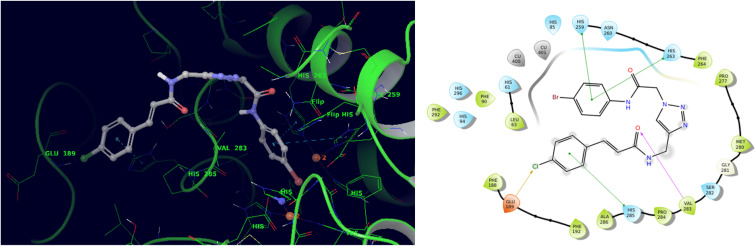
3D and 2D interaction pattern of 9i within the active site of tyrosinase.

Accordingly, molecular docking was performed to understand the binding pose and key interactions of compound 9i. The most potent compound demonstrated a docking energy of −8.082 kcal mol^−1^. As can be seen in Fig. 3, the 4-chlorophenyl group of 9i underwent π–π stacking interactions with His285, an important residue in the enzyme's active site. Conversely, the halogen bond was made with Glu189. The carbonyl oxygen of the cinnamic moiety formed a hydrogen bonding interaction with Val283. On the opposite side of the molecule, the 4-bromophenyl group engaged in two π–π stacking interactions with His263 and His259, both of which are known to be important residues for catalytic activity.

**Fig. 4:**
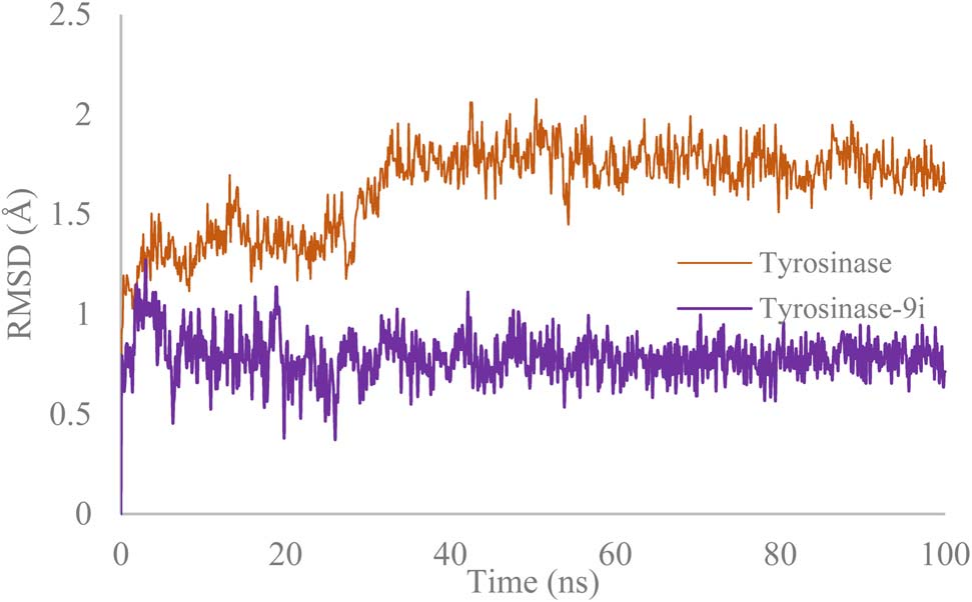
Superimposed RMSD of Cα atoms of tyrosinase in complex with compound 9i (purple) and tyrosinase (orange).

A molecular dynamics study was performed to compare the stability of the tyrosinase in complex with compound 9i against tyrosinase in its unbound state (Fig. 4). The RMSD of backbone atoms served as an indicator of stability for both systems. As illustrated in Fig. 4, the RMSD of 9i/tyrosinase was significantly lower (RMSD = 1 Å) compared to tyrosinase enzyme (RMSD = 2 Å), indicating stability of the complex over the 100 ns simulation.

**Fig. 5:**
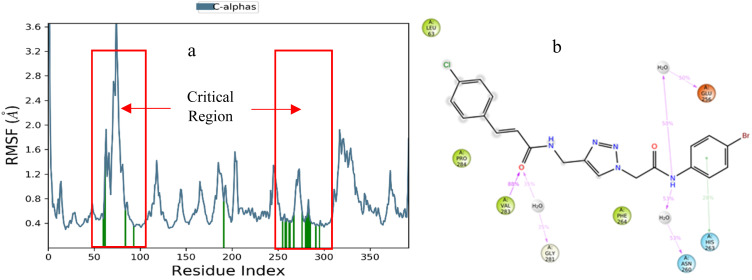
(a) RMSF of tyrosinase in complex with 9i (red). (b) Provides a schematic of detailed ligand interactions with protein residues occurring more than 30.0% of the simulation time.

The Root Mean Square Fluctuation (RMSF) plots of the protein were analyzed to assess the mobility and fluctuation of the protein's structure (Fig. 5a). The RMSF of 9i-tyrosinase enzyme exhibited low fluctuations at residues 59–97 and 254–297, known as the active site region. The interaction between the tyrosinase and the 9i ligand is visually represented in Fig. 5b, where these interactions were observed during MD simulation. The 4-bromophenyl amine moiety exhibited two hydrogen bonding interactions with Glu256 and Asn260 mediated with water, plus a π–π stacking interaction with His263 (also observed in molecular docking). Additionally, the cinnamic moiety also recorded one direct H-bonding interaction with Val293 and another with Val281, mediated by water (Fig. 5b).

### 2.6 Cell viability

Compound 9i, the compound that showed the most potent tyrosinase inhibitory activity, was then evaluated for cytotoxicity in the invasive melanoma B16F10 cells. The cells were then treated with varying concentrations of 9i, and their viability was assessed using the MTT assay. Concentrations below 100 µM were considered safe based on the melanin content assay (Table 3). At 100 µM, 9i maintained more than 80% viability, which indicates that this concentration is likely to be low in cytotoxicity.

**Table 3:** Cytotoxicity evaluation of 9i on the B16F10 cell line^a^

Concentration	% Viability ± SE^a^
200 µM	51.19 ± 4.34
150 µM	58.24 ± 3.43
100 µM	82.97 ± 5.31

a Data expressed as mean ± SE.

### 2.7 Melanin content assay

The effect of the most potent tyrosinase inhibitors on melanin content was analyzed in B16F10 melanoma cells. A melanin calibration curve was first plotted to find the concentration of melanin. Results showed that the untreated control cells produced 92 µg mL^−1^ of melanin. Treatment with 9i, on the contrary, decreased the melanin levels to 62 µg mL^−1^, meaning that there was a significant reduction in melanin content as compared to the control groups, thus affirming that 9i exhibits inhibitory potential to reduce melanin synthesis in B16F10 cells.

## 3. Conclusion

In this study, a series of novel aminomethyl 1,2,3-triazole–cinamamide hybrid compounds were synthesized and tested for their anti-tyrosinase, antioxidant, and anti-melanogenic effects. All compounds synthesized were screened for tyrosinase inhibition activity. The SAR investigations have evidenced that the substitution at R^1^ and R^2^ generally increases potency. Kinetic studies confirmed the competitive inhibition mechanism of 9i with a *K*_i_ of 34.36 µM, whereby it binds to the active site of the enzyme. The compounds exhibited mild to good antioxidant activities when examined by DPPH radical scavenging assays, which would help alleviate oxidative stress typically associated with hyperpigmentation disorders. The anti-melanogenic potential of these compounds was further corroborated through their biological activities on the B16F10 murine melanoma cell line. In particular, compound 9i significantly reduced melanin content to 62 µg mL^−1^ at 70 µM concentration compared with untreated controls. In contrast, the compound showed limited cytotoxicity, making it an ideal candidate for further drug development.

## 4. Method and materials

### 4.1 General procedure for the synthesis of 9a–q

A mixture of benzaldehyde derivatives (1a–c, 25 mmol), malonic acid (2, 25 mmol), pyridine (15 mL), and piperidine (5 mL) was stirred for 3 h at 100 °C. Thereafter, water (300 mL) was added, and the mixture was neutralized with concentrated HCl.^18^ To a mixture of cinnamic acid derivatives 3a–c (10 mmol) and propargylamine 4 (10 mmol) in DMF (50 mL), TBTU (13 mmol) and Et_3_N (13 mmol) were added, and the obtained mixture was stirred for 24 h at room temperature (rt). After completion of the reaction, water was added to this reaction mixture, and the precipitate was filtered to give pure *N*-(prop-2-yn-1-yl)cinnamamide derivatives 5a–c.^19^

Aniline derivatives (6a–e, 50 mmol) were suspended in DMF (25 mL) and cooled to 0 °C. Next, chloroacetyl chloride (65 mmol) was added, and the reaction mixture was then stirred at room temperature for 24 h. At the end of the reaction, the reaction mixture was diluted with water, poured into crushed ice, and the resulting precipitates were filtered off. Finally, the residue was washed with water to obtain 2-chloro-*N*-substituted acetamide derivatives 7a–e.^20,21^ A mixture of acetamides 7a–o (1.2 mmol), K_2_CO_3_ (1.5 mmol), and NaN_3_ (1.5 mmol) in DMF was stirred at room temperature for 1 h. Then, copper sulfate pentahydrate (CuSO4·5H_2_O, 0.15 mmol), sodium ascorbate (0.45 mmol), and *N*-(prop-2-yn-1-yl)cinnamamide derivatives (5a–c, 1 mmol) were added to the reaction mixture. After the completion of the reaction, copper ions were removed from the reaction mixture using an EDTA (ethylenediaminetetraacetic acid) solution (0.05 M). Then, the resulting precipitate was filtered and washed several times with water and crystallized from ethyl acetate to obtain pure *N*-((1*H*-1,2,3-triazol-4-yl)methyl)cinnamamide-*N*-substituted acetamides 9a–q.

#### 4.1.1 *N*-((1-(2-Oxo-2-(phenylamino)ethyl)-1*H*-1,2,3-triazol-4-yl)methyl)cinnamamide (9a)

Brown solid; yield: 89%; mp = 141–143 °C; IR (KBr, *v*_max_): 3295, 3010, 2935, 1658 cm^−1^. ^1^H NMR (300 MHz, DMSO-*d*_6_): *δ* 10.51 (s, 1H, NH), 8.74 (t, *J* = 5.0 Hz, 1H, NH), 8.07 (s, 1H, triazole), 7.66–7.57 (m, 4H, Ar), 7.53 (d, *J* = 15.8 Hz, 1H, CH), 7.46–7.30 (m, 5H, Ar), 7.10 (t, *J* = 7.3 Hz, 1H, Ar), 6.74 (d, *J* = 15.8 Hz, 1H, CH), 5.36 (s, 2H, CH_2_), 4.53 (d, *J* = 5.3 Hz, 2H, CH_2_). ^13^C NMR (75 MHz, DMSO-*d*_6_): *δ* 165.4, 164.7, 145.3, 139.6, 138.9, 135.3, 132.3, 130.0, 129.4, 128.0, 125.2, 124.2, 122.4, 119.7, 52.7, 34.8. C_20_H_19_N_5_O_2_; calcd C, 66.47; H, 5.30; N, 19.38; found C, 66.62; H, 5.48; N, 19.56.

#### 4.1.2 *N*-((1-(2-((4-Fluorophenyl)amino)-2-oxoethyl)-1*H*-1,2,3-triazol-4-yl)methyl)cinnamamide (9b)

Brown solid; yield: 69%; mp = 168–170 °C; IR (KBr, *v*_max_): 3347, 3030, 2950, 1666 cm^−1^. ^1^H NMR (300 MHz, DMSO-*d*_6_): *δ* 10.56 (s, 1H, NH), 8.73 (t, *J* = 5.5 Hz, 1H, NH), 8.05 (s, 1H, triazole), 7.67–7.56 (m, 4H, Ar), 7.52 (d, *J* = 15.8 Hz, 1H, CH), 7.47–7.37 (m, 3H, CH), 7.19 (t, *J* = 8.8 Hz, 2H, Ar), 6.73 (d, *J* = 15.8 Hz, 1H, CH), 5.35 (s, 2H, CH_2_), 4.52 (d, *J* = 5.5 Hz, 2H, CH_2_). ^13^C NMR (75 MHz, DMSO-*d*_6_): *δ* 165.4, 164.7, 158.7 (d, *J* = 240.3 Hz), 145.2, 139.5, 135.3, 135.2, 130.0, 129.4, 128.0, 125.1, 122.4, 121.5 (d, *J* = 7.9 Hz), 116.0 (d, *J* = 22.1 Hz), 52.58, 34.8. C_20_H_18_FN_5_O_2_; calcd C, 63.32; H, 4.78; N, 18.46; found C, 63.47; H, 4.93; N, 18.68.

#### 4.1.3 *N*-((1-(2-((4-Chlorophenyl)amino)-2-oxoethyl)-1*H*-1,2,3-triazol-4-yl)methyl)cinnamamide (9c)

Brown solid; yield: 78%; mp = 152–154 °C; IR (KBr, *v*_max_): 3320, 3035, 2955, 1664 cm^−1^. ^1^H NMR (300 MHz, DMSO-*d*_*6*_): *δ* 10.63 (s, 1H, NH), 8.71 (t, *J* = 5.5 Hz, 1H, NH), 8.04 (s, 1H, triazole), 7.64 (d, *J* = 8.8 Hz, 2H, Ar), 7.61–7.55 (m, 2H, Ar), 7.50 (d, *J* = 15.8 Hz, 1H, CH), 7.47–7.33 (m, 5H, Ar), 6.72 (d, *J* = 15.8 Hz, 1H, CH), 5.35 (s, 2H, CH_2_), 4.50 (d, *J* = 5.5 Hz, 2H, CH_2_). ^13^C NMR (75 MHz, DMSO-*d*_6_): *δ* 165.4, 164.9, 145.2, 139.5, 137.9, 135.3, 130.0, 129.4, 129.3, 128.0, 127.8, 125.1, 122.4, 121.2, 52.6, 34.8. C_20_H_18_ClN_5_O_2_; calcd C, 60.69; H, 4.58; N, 17.69; found C, 60.86; H, 4.76; N, 17.89.

#### 4.1.4 *N*-((1-(2-((4-Bromophenyl)amino)-2-oxoethyl)-1*H*-1,2,3-triazol-4-yl)methyl)cinnamamide (9d)

Brown solid; yield: 65%; mp = 161–163 °C; IR (KBr, *v*_max_): 3334, 304, 2960, 1667 cm^−1^. ^1^H NMR (300 MHz, DMSO-*d*_*6*_): *δ* 10.64 (s, 1H, NH), 8.72 (t, *J* = 4.8 Hz, 1H, NH), 8.05 (s, 1H, triazole), 7.61–7.51 (m, 6H, Ar), 7.50–7.36 (m, 4H, Ar, CH), 6.72 (d, *J* = 15.8 Hz, 1H, CH), 5.35 (s, 2H, CH_2_), 4.50 (d, *J* = 4.8 Hz, 2H, CH_2_). ^13^C NMR (75 MHz, DMSO-*d*_6_): *δ* 165.4, 165.0, 145.1, 139.5, 138.3, 135.3, 132.2, 130.0, 129.4, 128.0, 125.1, 122.4, 121.6, 115.9, 52.6, 34.8. C_20_H_18_BrN_5_O_2_; calcd C, 54.56; H, 4.12; N, 15.91; found C, 54.56; H, 4.12; N, 15.91.

#### 4.1.5 *N*-((1-(2-((4-Ethylphenyl)amino)-2-oxoethyl)-1*H*-1,2,3-triazol-4-yl)methyl)cinnamamide (9e)

Brown solid; yield: 68%; mp = 158–160 °C; IR (KBr, *v*_max_): 3311, 3020, 2950, 1663 cm^−1^; ^1^H NMR (300 MHz, DMSO-*d*_6_): *δ* 10.44 (s, 1H, NH), 8.73 (t, *J* = 5.3 Hz, 1H, NH), 8.05 (s, 1H, triazole), 7.64–7.48 (m, 5H, Ar), 7.46–7.37 (m, 3H, Ar, CH), 7.18 (d, *J* = 7.5 Hz, 2H, Ar), 6.74 (d, *J* = 15.8 Hz, 1H, CH), 5.34 (s, 2H, CH_2_), 4.52 (d, *J* = 5.3 Hz, 2H, CH_2_), 2.57 (q, *J* = 7.4 Hz, 2H, CH_2_), 1.17 (t, *J* = 7.4 Hz, 3H, CH_3_). ^13^C NMR (75 MHz, DMSO-*d*_6_): *δ* 165.4, 164.5, 145.2, 139.7, 139.5, 136.6, 135.3, 130.0, 129.4, 128.6, 128.0, 125.1, 122.4, 119.8, 52.6, 34.8, 28.1, 16.1. C_22_H_23_N_5_O_2_; calcd C, 67.85; H, 5.95; N, 17.98; found C, 68.02; H, 6.12; N, 18.15.

#### 4.1.6 3-(4-Chlorophenyl)-*N*-((1-(2-oxo-2-(phenylamino)ethyl)-1*H*-1,2,3-triazol-4-yl)methyl)acrylamide (9f)

Brown solid; yield: 73%; mp = 163–165 °C; IR (KBr, *v*_max_): 3337, 3030, 2940, 1661 cm^−1^. ^1^H NMR (300 MHz, DMSO-*d*_6_): *δ* 10.50 (s, 1H, NH), 8.74 (t, *J* = 5.3 Hz, 1H, NH), 8.05 (s, 1H, triazole), 7.64–7.57 (m, 4H, Ar), 7.54–7.45 (m, 3H, Ar, CH), 7.40–7.30 (m, 2H, Ar), 7.10 (t, *J* = 7.2 Hz, 1H, Ar), 6.73 (d, *J* = 15.8 Hz, 1H, CH), 5.35 (s, 2H, CH_2_), 4.51 (d, *J* = 5.3 Hz, 2H, CH_2_). ^13^C NMR (75 MHz, DMSO-*d*_6_) *δ* 165.2, 164.7, 145.0, 138.9, 138.2, 134.4, 134.3, 129.7, 129.4, 129.4, 125.1, 124.3, 123.1, 119.7, 52.6, 34.8. C_20_H_18_ClN_5_O_2_; calcd C, 60.69; H, 4.58; N, 17.69; found C, 60.78; H, 4.70; N, 17.91.

#### 4.1.7 3-(4-Chlorophenyl)-*N*-((1-(2-((4-fluorophenyl)amino)-2-oxoethyl)-1*H*-1,2,3-triazol-4-yl)methyl)acrylamide (9g)

Brown solid; yield: 60%; mp = 185–187 °C; IR (KBr, *v*_max_): 3351, 3025, 2965, 1668 cm^−1^. ^1^H NMR (300 MHz, DMSO-*d*_6_): *δ* 10.56 (s, 1H, NH), 8.73 (t, *J* = 5.0 Hz, 1H, NH), 8.05 (s, 1H, triazole), 7.71–7.57 (m, 4H, Ar), 7.54–7.40 (m, 3H, Ar, CH), 7.23–7.11 (m, 2H, Ar), 6.72 (d, *J* = 15.8 Hz, 1H, CH), 5.34 (s, 2H, CH_2_), 4.51 (d, *J* = 5.0 Hz, 2H, CH_2_). ^13^C NMR (76 MHz, DMSO-*d*_6_): *δ* 165.2, 164.7, 158.7 (d, *J* = 240.3 Hz), 145.0, 138.2, 135.3, 134.3 (d, *J* = 9.3 Hz), 129.7, 129.4, 125.1, 123.2, 121.5, 121.4, 116.0 (d, *J* = 22.1 Hz), 52.6, 34.8. C_20_H_17_ClFN_5_O_2_; calcd C, 58.05; H, 4.14; N, 16.92; found C, 58.19; H, 4.31; N, 17.10.

#### 4.1.8 3-(4-Chlorophenyl)-*N*-((1-(2-((4-chlorophenyl)amino)-2-oxoethyl)-1*H*-1,2,3-triazol-4-yl)methyl)acrylamide (9h)

Brown solid; yield: 58%; mp = 174–1176 °C; IR (KBr, *v*_max_): 3342, 3055, 2945, 1667 cm^−1^. ^1^H NMR (300 MHz, DMSO-*d*_6_): *δ* 10.64 (s, 1H, NH), 8.74 (t, *J* = 5.2 Hz, 1H, NH), 8.04 (s, 1H, triazole), 7.66–7.58 (m, 4H, Ar), 7.54–7.45 (m, 3H, Ar, CH), 7.40 (d, *J* = 8.7 Hz, 2H, Ar), 6.72 (d, *J* = 15.8 Hz, 1H, CH), 5.35 (s, 2H, CH_2_), 4.50 (d, *J* = 5.2 Hz, 2H, CH_2_). ^13^C NMR (75 MHz, DMSO-*d*_6_): *δ* 165.2, 164.9, 145.2, 138.2, 137.8, 134.4, 134.3, 129.7, 129.4, 129.3, 127.9, 125.2, 123.1, 121.3, 52.6, 34.8. C_20_H_17_C_l2_N_5_O_2_; calcd C, 55.83; H, 3.98; N, 16.28; found C, 55.98; H, 4.16; N, 16.39.

#### 4.1.9 *N*-((1-(2-((4-Bromophenyl)amino)-2-oxoethyl)-1*H*-1,2,3-triazol-4-yl)methyl)-3-(4-chlorophenyl)acrylamide (9i)

Brown solid; yield: 61%; mp = 161–163 °C; IR (KBr, *v*_max_): 3352, 3060, 2935, 1665 cm^−1^. ^1^H NMR (300 MHz, DMSO-*d*_6_): *δ* 10.63 (s, 1H, NH), 8.73 (t, *J* = 5.0 Hz, 1H, NH), 8.03 (s, 1H, triazole), 7.63–7.58 (m, 3H, Ar), 7.57–7.53 (m, 3H, Ar), 7.52–7.45 (m, 3H, Ar, CH), 6.71 (d, *J* = 15.8 Hz, 1H, CH), 5.34 (s, 2H, CH_2_), 4.49 (d, *J* = 5.0 Hz, 2H, CH_2_). ^13^C NMR (75 MHz, DMSO-*d*_6_): *δ* 165.2, 164.9, 145.1, 138.2, 138.2, 134.4, 134.3, 132.2, 129.7, 129.5, 125.1, 123.2, 121.6, 115.9, 52.6, 34.8. C_20_H_17_BrClN_5_O_2_; calcd C, 50.60; H, 3.61; N, 14.75; found C, 50.74; H, 3.82; N, 14.88. The purity of the compound was checked using HPLC.

#### 4.1.10 3-(4-Chlorophenyl)-*N*-((1-(2-((4-ethylphenyl)amino)-2-oxoethyl)-1*H*-1,2,3-triazol-4-yl)methyl)acrylamide (9j)

Brown solid; yield: 59%; mp = 172–174 °C; IR (KBr, *v*_max_): 3322, 3040, 2965, 1668 cm^−1^. ^1^H NMR (300 MHz, DMSO-*d*_6_): *δ* 10.41 (s, 1H, NH), 8.73 (t, *J* = 5.4 Hz, 1H, NH), 8.04 (s, 1H, triazole), 7.61 (d, *J* = 8.5 Hz, 2H, Ar), 7.56–7.45 (m, 5H, Ar, CH), 7.17 (d, *J* = 8.5 Hz, 2H, Ar), 6.73 (d, *J* = 15.8 Hz, 1H, CH), 5.33 (s, 2H, CH_2_), 4.51 (d, *J* = 5.4 Hz, 2H, CH_2_), 2.55 (q, *J* = 7.6 Hz, 2H, CH_2_), 1.16 (t, *J* = 7.6 Hz, 3H, CH_3_). ^13^C NMR (75 MHz, DMSO-*d*_6_): *δ* 165.2, 164.5, 145.0, 139.6, 138.2, 136.6, 134.4, 134.3, 129.7, 129.4, 128.6, 125.1, 123.2, 119.8, 52.63, 34.82, 28.07, 16.10. C_22_H_22_ClN_5_O_2_; calcd C, 62.34; H, 5.23; N, 16.52; found C, 62.51; H, 5.38; N, 16.73.

#### 4.1.11 3-(4-Chlorophenyl)-*N*-((1-(2-((4-methoxyphenyl)amino)-2-oxoethyl)-1*H*-1,2,3-triazol-4-yl)methyl)acrylamide (9k)

Brown solid; yield: 79%; mp = 182–184 °C; IR (KBr, *v*_max_): 3336, 3015, 2970, 1673 cm^−1^. ^1^H NMR (400 MHz, DMSO-*d*_6_) *δ* 10.34 (s, 1H, NH), 8.71 (t, *J* = 5.8 Hz, 1H, NH), 8.01 (s, 1H, triazole), 7.60 (d, *J* = 8.5 Hz, 2H, Ar), 7.53–7.42 (m, 5H, Ar, CH), 6.91 (d, *J* = 9.0 Hz, 2H, Ar), 6.70 (d, *J* = 15.8 Hz, 1H, CH), 5.28 (s, 2H, CH_2_), 4.48 (d, *J* = 5.8 Hz, 2H, CH_2_), 3.72 (s, 3H, OCH_3_). ^13^C NMR (100 MHz, DMSO-*d*_6_) *δ* 165.1, 164.2, 156.0, 145.0, 138.2, 134.4, 134.3, 132.0, 129.7, 129.5, 125.1, 123.2, 121.2, 114.5, 55.6, 52.5, 34.8. C_21_H_20_ClN_5_O_3_; calcd C, 59.23; H, 4.73; N, 16.45; found C, 59.47; H, 4.88; N, 16.59.

#### 4.1.12 3-(4-Methoxyphenyl)-*N*-((1-(2-oxo-2-(phenylamino)ethyl)-1*H*-1,2,3-triazol-4-yl)methyl)acrylamide (9l)

Brown solid; yield: 81%; mp = 159–161 °C; IR (KBr, *v*_max_): 3319, 3045, 2950, 1670 cm^−1^. ^1^H NMR (300 MHz, DMSO-*d*_6_): *δ* 10.52 (s, 1H, NH), 8.64 (t, *J* = 5.0 Hz, 1H, NH), 8.05 (s, 1H, triazole), 7.62 (d, *J* = 7.5 Hz, 2H, Ar), 7.54 (d, *J* = 8.4 Hz, 2H, Ar), 7.47 (d, *J* = 15.7 Hz, 1H, CH), 7.35 (t, *J* = 7.5 Hz, 2H, Ar), 7.10 (t, *J* = 7.5 Hz, 1H, Ar), 6.99 (d, *J* = 8.4 Hz, 2H, Ar), 6.59 (d, *J* = 15.7 Hz, 1H, CH), 5.36 (s, 2H, CH_2_), 4.51 (d, *J* = 5.0 Hz, 2H, CH_2_), 3.79 (s, 3H, OCH_3_). ^13^C NMR (75 MHz, DMSO-*d*_6_): *δ* 165.7, 164.7, 160.8, 145.2, 139.2, 138.9, 129.6, 129.4, 127.9, 125.1, 124.2, 119.9, 119.7, 114.9, 55.7, 52.7, 34.8. C_21_H_21_N_5_O_3_; calcd C, 64.44; H, 5.41; N, 17.89; found C, 64.57; H, 5.64; N, 18.07.

#### 4.1.13 *N*-((1-(2-((4-Fluorophenyl)amino)-2-oxoethyl)-1*H*-1,2,3-triazol-4-yl)methyl)-3-(4-methoxyphenyl)acrylamide (9m)

Brown solid; yield: 56%; mp = 187–189 °C; IR (KBr, *v*_max_): 3337, 3055, 2965, 1668 cm^−1^. ^1^H NMR (300 MHz, DMSO-*d*_6_): *δ* 10.58 (s, 1H, NH), 8.63 (t, *J* = 5.0 Hz, 1H, NH), 8.04 (s, 1H, triazole), 7.69–7.59 (m, 3H, Ar), 7.53 (d, *J* = 8.5 Hz, 2H, Ar), 7.46 (d, *J* = 15.7 Hz, 1H, CH), 7.19 (t, *J* = 8.7 Hz, 2H, Ar), 6.99 (d, *J* = 8.5 Hz, 2H, Ar), 6.58 (d, *J* = 15.8 Hz, 1H, CH), 5.35 (s, 2H, CH_2_), 4.50 (d, *J* = 5.0 Hz, 2H, CH_2_), 3.79 (s, 3H, OCH_3_). ^13^C NMR (75 MHz, DMSO-*d*_6_): *δ* 165.7, 164.7, 160.8, 158.7 (d, *J* = 240.4 Hz), 145.2, 139.2, 135.3 (d, *J* = 2.5 Hz), 129.6, 127.9, 125.1, 121.5, 119.9, 116.0 (d, *J* = 22.1 Hz), 114.8, 55.69, 52.58, 34.78. C_21_H_20_FN_5_O_3_; calcd C, 61.61; H, 4.92; N, 17.11; found C, 61.78; H, 5.09; N, 17.34.

#### 4.1.14 *N*-((1-(2-((4-Chlorophenyl)amino)-2-oxoethyl)-1*H*-1,2,3-triazol-4-yl)methyl)-3-(4-methoxyphenyl)acrylamide (9n)

Brown solid; yield: 68%; mp = 179–181 °C; IR (KBr, *v*_max_): 3338, 3020, 2960, 1671 cm^−1^. ^1^H NMR (300 MHz, DMSO-*d*_6_): *δ* 10.64 (s, 1H, NH), 8.63 (t, *J* = 5.4 Hz, 1H, NH), 8.05 (s, 1H, triazole), 7.64 (d, *J* = 8.8 Hz, 2H), 7.53 (d, *J* = 8.6 Hz, 2H), 7.47 (d, *J* = 15.8 Hz, 1H), 7.40 (d, *J* = 8.8 Hz, 2H), 6.98 (d, *J* = 8.6 Hz, 2H), 6.58 (d, *J* = 15.8 Hz, 1H), 5.36 (s, 2H, CH_2_), 4.51 (d, *J* = 5.4 Hz, 2H, CH_2_), 3.79 (s, 3H, OCH_3_). ^13^C NMR (75 MHz, DMSO-*d*_6_): *δ* 165.8, 164.9, 160.8, 145.3, 139.3, 137.8, 131.9, 129.6, 129.3, 127.9, 125.1, 121.3, 119.9, 114.8, 55.7, 52.6, 34.8. C_21_H_20_ClN_5_O_3_; calcd C, 59.23; H, 4.73; N, 16.45; found C, 59.48; H, 4.86; N, 16.61.

#### 4.1.15 *N*-((1-(2-((4-Bromophenyl)amino)-2-oxoethyl)-1*H*-1,2,3-triazol-4-yl)methyl)-3-(4-methoxyphenyl)acrylamide (9o)

Brown solid; yield: 68%; mp = 180–182 °C; IR (KBr, *v*_max_): 3349, 3065, 2935, 1674 cm^−1^. ^1^H NMR (300 MHz, DMSO-*d*_6_): *δ* 10.63 (s, 1H, NH), 8.61 (t, *J* = 5.6 Hz, 1H, NH), 8.03 (s, 1H, triazole), 7.62–7.50 (m, 6H, Ar), 7.46 (d, *J* = 15.8 Hz, 1H, CH), 6.99 (d, *J* = 8.7 Hz, 2H, Ar), 6.57 (d, *J* = 15.8 Hz, 1H, CH), 5.35 (s, 2H, CH_2_), 4.50 (d, *J* = 5.6 Hz, 2H, CH_2_), 3.80 (s, 3H, OCH_3_). ^13^C NMR (75 MHz, DMSO-*d*_6_): *δ* 165.7, 165.0, 160.8, 145.2, 139.2, 138.2, 132.2, 129.6, 127.9, 125.1, 121.6, 119.9, 115.9, 114.9, 55.7, 52.6, 34.8. C_21_H_20_BrN_5_O_3_; calcd C, 53.63; H, 4.29; N, 14.89; found C, 53.78; H, 4.46; N, 15.04.

#### 4.1.16 *N*-((1-(2-((4-Ethylphenyl)amino)-2-oxoethyl)-1*H*-1,2,3-triazol-4-yl)methyl)-3-(4 methoxyphenyl)acrylamide (9p)

Brown solid; yield: 74%; mp = 166–168 °C; IR (KBr, *v*_max_): 3333, 3050, 2935, 1669 cm^−1^. ^1^H NMR (300 MHz, DMSO-*d*_6_): *δ* 10.44 (s, 1H, NH), 8.64 (t, *J* = 5.3 Hz, 1H, NH), 8.05 (s, 1H, triazole), 7.59–7.41 (m, 5H, Ar, CH), 7.17 (d, *J* = 7.8 Hz, 2H, Ar), 6.99 (d, *J* = 8.1 Hz, 2H, Ar), 6.60 (d, *J* = 15.7 Hz, 1H, CH), 5.34 (s, 2H, CH_2_), 4.51 (d, *J* = 5.3 Hz, 2H, CH_2_), 3.79 (s, 3H, OCH_3_), 2.55 (*q* = 7.5 Hz, 2H, CH_2_), 1.16 (t, *J* = 7.5 Hz, 3H, CH_3_). ^13^C NMR (75 MHz, DMSO-*d*_6_): *δ* 165.7, 164.5, 160.8, 145.2, 139.7, 139.3, 136.6, 129.6, 128.6, 127.9, 125.1, 119.9, 119.8, 114.4, 55.7, 52.6, 34.8, 28.1, 16.1. C_23_H_25_N_5_O_3_; calcd C, 65.86; H, 6.01; N, 16.70; found C, 66.03; H, 6.17; N, 16.91.

#### 4.1.17 3-(4-Methoxyphenyl)-*N*-((1-(2-((4-methoxyphenyl)amino)-2-oxoethyl)-1*H*-1,2,3-triazol-4-yl)methyl)acrylamide (9q)

Brown solid; yield: 87%; mp = 173–175 °C; IR (KBr, *v*_max_): 3324, 3032, 2945, 1670 cm^−1^. ^1^H NMR (400 MHz, DMSO-*d*_6_) *δ* 10.34 (s, 1H, NH), 8.59 (t, *J* = 5.8 Hz, 1H, NH), 8.00 (s, 1H, triazole), 7.57–7.48 (m, 4H, Ar), 7.43 (d, *J* = 15.8 Hz, 1H, CH), 6.98 (d, *J* = 8.5 Hz, 2H, Ar), 6.91 (d, *J* = 9.1 Hz, 2H, Ar), 6.55 (d, *J* = 15.7 Hz, 1H, CH), 5.28 (s, 2H, CH_2_), 4.47 (d, *J* = 5.7 Hz, 2H, CH_2_), 3.78 (s, 3H, OCH_3_), 3.72 (s, 3H, OCH_3_). ^13^C NMR (100 MHz, DMSO-*d*_6_) *δ* 165.7, 164.2, 160.8, 156.0, 145.2, 139.2, 132.0, 129.6, 127.9, 125.0, 121.2, 119.9, 114.8, 114.5, 55.7, 55.6, 52.5, 34.7. C_22_H_23_N_5_O_4_; calcd C, 62.70; H, 5.50; N, 16.62; found C, 63.03; H, 5.65; N, 16.81. MS ES^+^: *m*/*z* 442.2910 [M + 1]^+^ and 445.2930 [M + 23]^+^.

### 4.2 Tyrosinase inhibitory assay

The tyrosinase activity assay for compounds followed a modified procedure. In brief, in a 96-well microplate, 160 µL of phosphate buffer (50 mM, pH = 6.8), 10 µL of lyophilized mushroom tyrosinase powder (CAS no. 9002-10-2 dissolved in water with 500 units per mg), and 10 µL of the test compound (dissolved in DMSO) were added. Subsequently, 20 µL of l-DOPA (dissolved in water) was added to initiate the enzymatic reaction. The change in absorbance at 475 nm was continuously monitored using a spectrophotometer. DMSO without test compounds served as the control, while kojic acid acted as the positive control.^22,23^

### 4.3 Determination of the inhibition type

The most potent derivative was selected for kinetic analysis, and its inhibitory activity was assessed at different inhibitor concentrations. The substrate (l-DOPA) concentrations ranged from 0.5 to 2.8 mM according to the previously reported procedures.^24^

### 4.4 Free radical-scavenging activity evaluation

As described previously, radical-scavenging activity was assessed using the 2,2-diphenyl-1-picrylhydrazyl (DPPH) assay. Briefly, the mixture of different concentrations of the compounds and DPPH methanolic solution (110 µM) was shaken in the dark at room temperature for 30 min. The mixture absorbance was measured at 517 nm. Quercetin was used as the positive control.^25^

### 4.5 Molecular docking study

Docking analysis was conducted to elucidate the interaction modes of the designed molecules with the tyrosinase enzyme. The Maestro Molecular Modeling by Schrödinger was utilized for this purpose. The X-ray crystal structure of the receptor (PDB ID: 2Y9X), with tropolone, was extracted from the Protein Data Bank. Before docking, protein preparation involved the removal of the native ligand and water molecules, followed by the addition of hydrogen atoms, with nonpolar hydrogens subsequently merged. The 2D structures of all synthesized compounds were drawn using the Marvin program, and their 3D structures were generated and optimized using LigPrep.^26^

### 4.6 Molecular dynamics simulation

MD simulation was conducted using Desmond v5.3 (part of the Schrödinger 2018-4 suite) on the best pose of the 9i complex extracted from induced fit docking.^27^

### 4.7 MTT assay for cell viability

The cytotoxic activity of all derivatives was determined using the 3-(4,5-dimethylthiazol-2-yl)-2,5-diphenyltetrazolium bromide (MTT) assay. B16F10 were obtained from the Pasteur Institute of Iran (https://en.pasteur.ac.ir/). Cells (at a density of 25 000 cells per ml) were grown at 37 °C in the presence of CO_2_ 5% in DMEM (Gibco BRL, Grand Island, NY, USA), 10% fetal bovine serum (FBS, Gibco BRL). Next, cells were seeded in a 96-well plate and incubated at 37 °C with the derivative at different concentrations for 72 h. Following treatment, cells were incubated with MTT (0.5 mg mL^−1^) for 3 h. The MTT-containing medium was then removed, and 100 µL of DMSO was added to each well, mixed thoroughly with a 10-minute shake to dissolve the formazan crystals. The absorbance of each well was measured at 540 nm.^28^

### 4.8 Determination of melanin content

The melanin content assay was performed according to previously reported protocols with minor modifications. Briefly, B16F10 cells were seeded in 12-well plates at a density of 50 000 cells per well. After 24 hours of incubation, 100 nM α-MSH was added to each well, followed by the addition of compound 9i five hours later. The plates were then incubated for an additional 48 hours. Subsequently, the cells were washed twice with PBS and harvested using 0.25 M trypsin. The cell pellets were dissolved in 200 µL of 1 N NaOH containing 10% DMSO and incubated at 80 °C for 2 hours to solubilize the melanin. The absorbance of the resulting supernatant was measured at 470 nm using a microplate reader. The melanin content was quantified using a calibration curve constructed from synthetic melanin standards, measured at 405 nm. Kojic acid was used as a positive control for comparison.^29^

## Conflicts of interest

There are no conflicts to declare.

## Supplementary Material

RA-015-D5RA04315H-s001

## Data Availability

The datasets generated and/or analyzed during the current study are available in the Worldwide Protein Data Bank under the PDB ID 2Y9X (https://www.rcsb.org/structure/2Y9X). The NMR and spectral data are provided in the supplementary information (SI). Supplementary information is available. See DOI: https://doi.org/10.1039/d5ra04315h.
