# Supplementary material for: Enzymatic, cell-based, and in silico evaluation of di-substituted aminomethyl-1,2,3-triazole–cinamamide hybrids as mushroom tyrosinase inhibitors
Source: RSC Adv. 2025 Oct 13;15(45):38014–23. doi: 10.1039/d5ra04315h (PMC12516498; doi:10.1039/d5ra04315h)

This journal is © The Royal Society of Chemistry 2025

Fig. S1. NMR of 9a

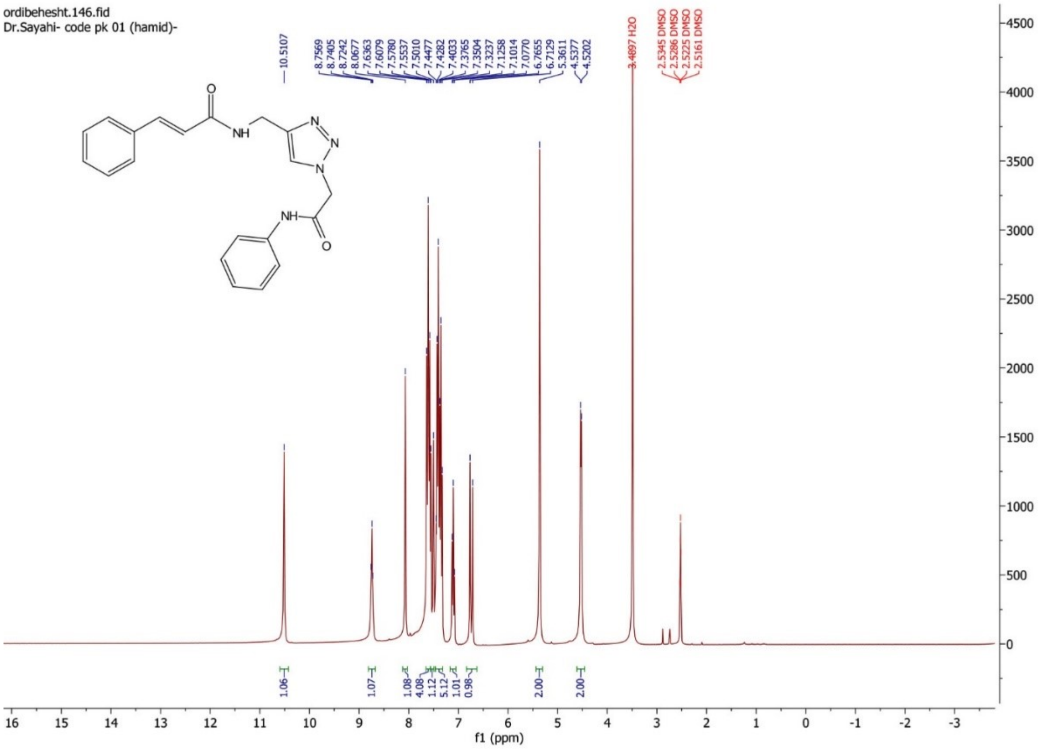

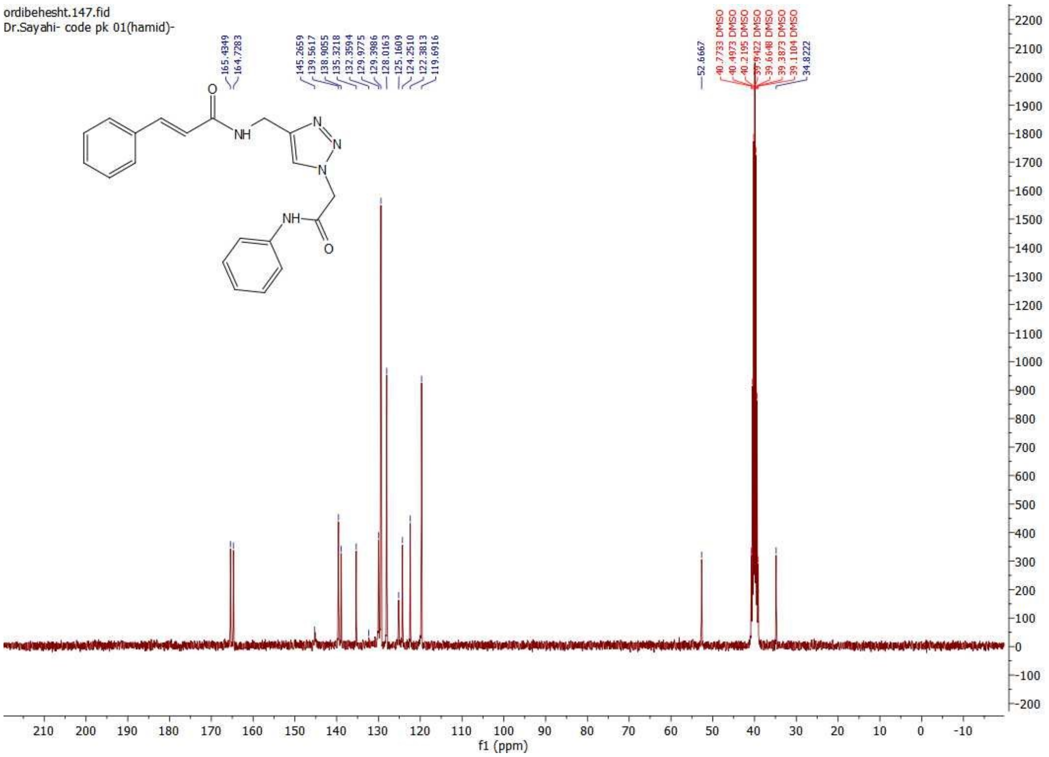

Fig. S2. NMR of 9b

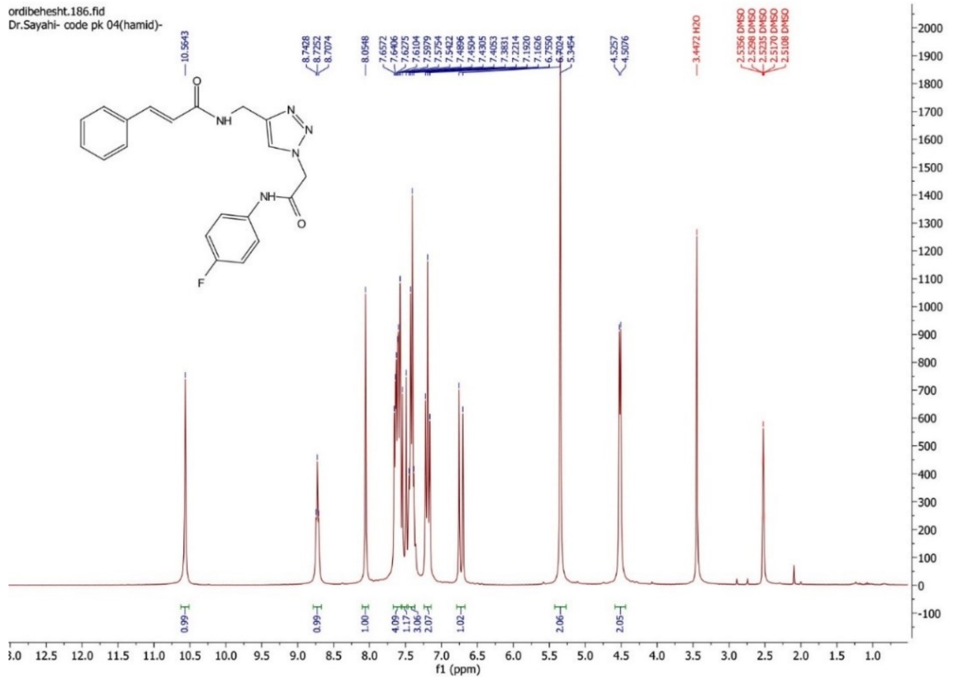

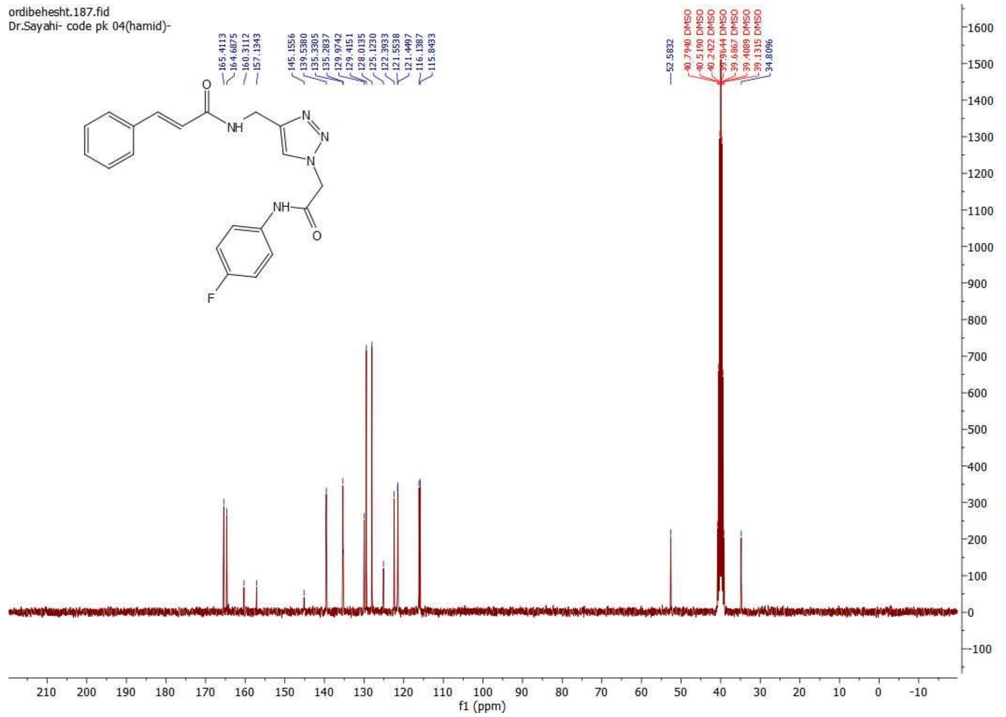

Fig. S3. NMR of 9c

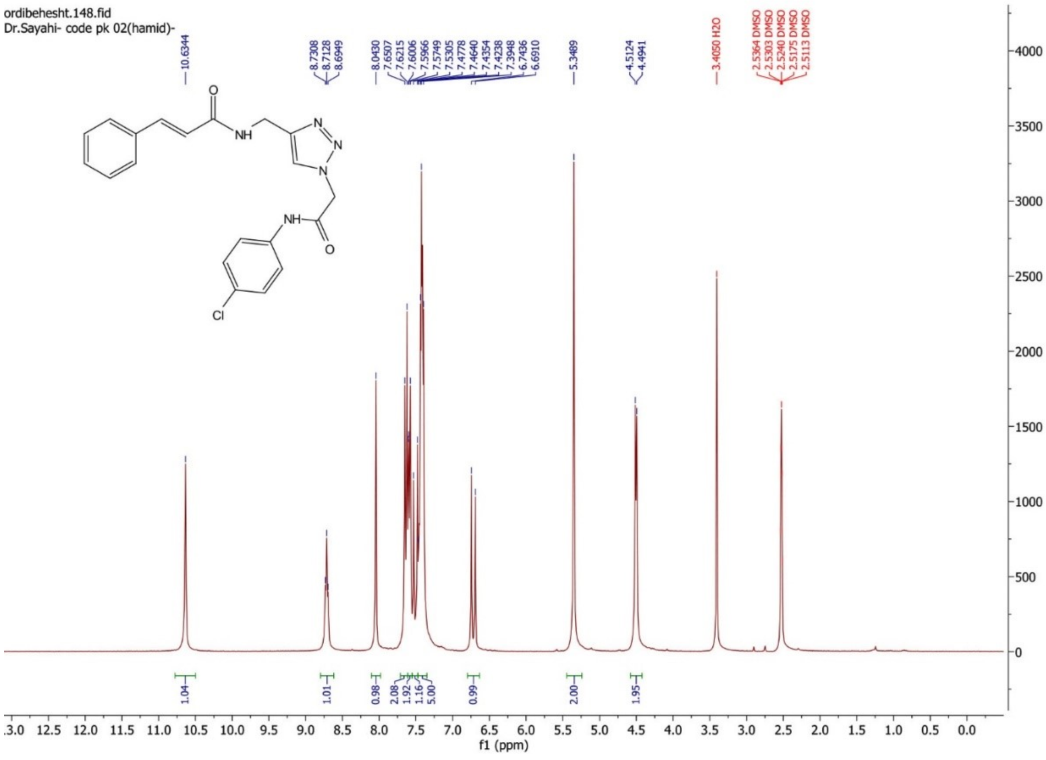

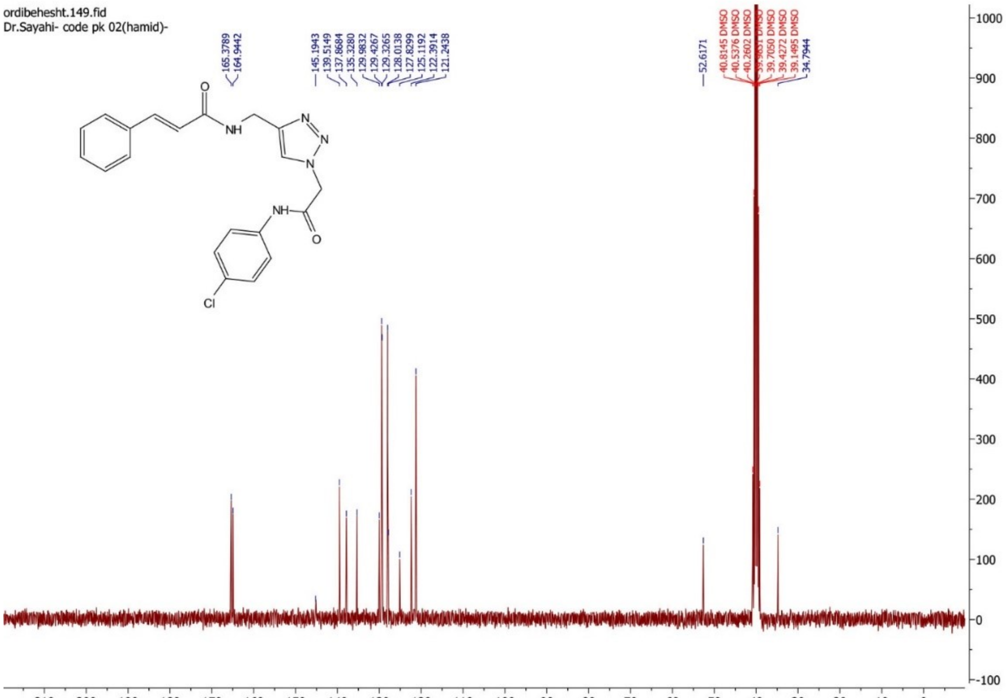

Fig. S4. NMR of 9d

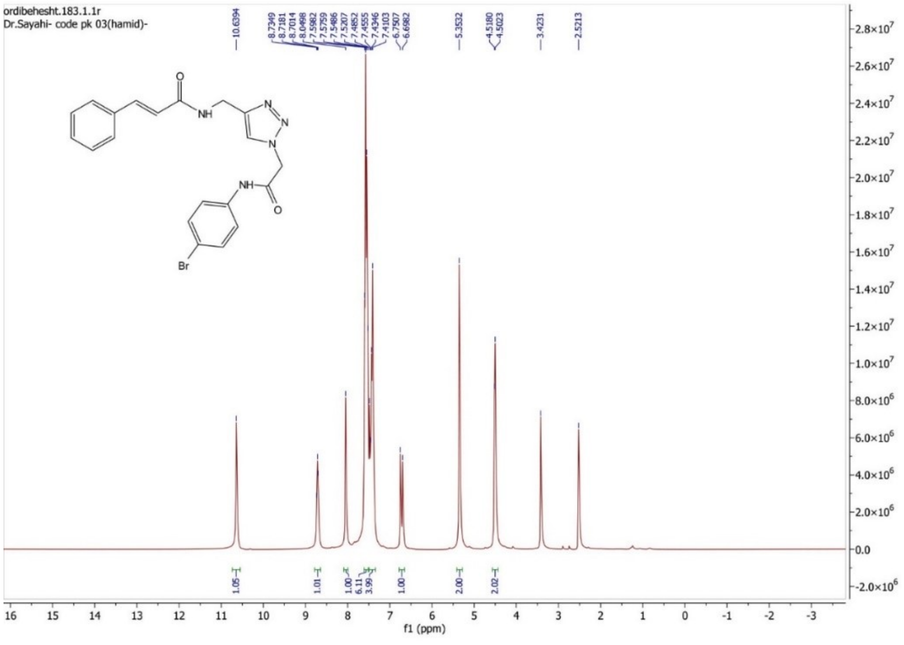

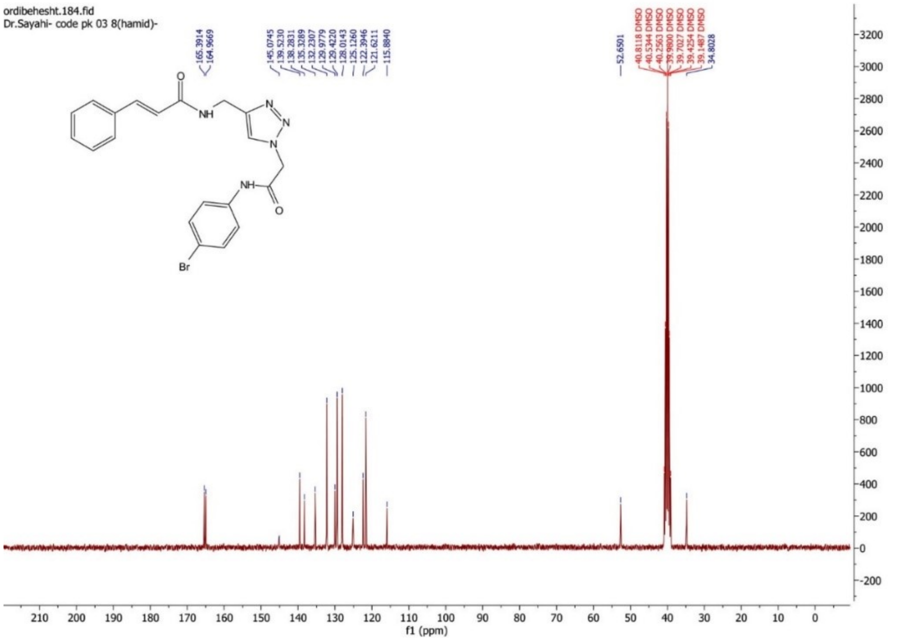

Fig. S5. NMR of 9e

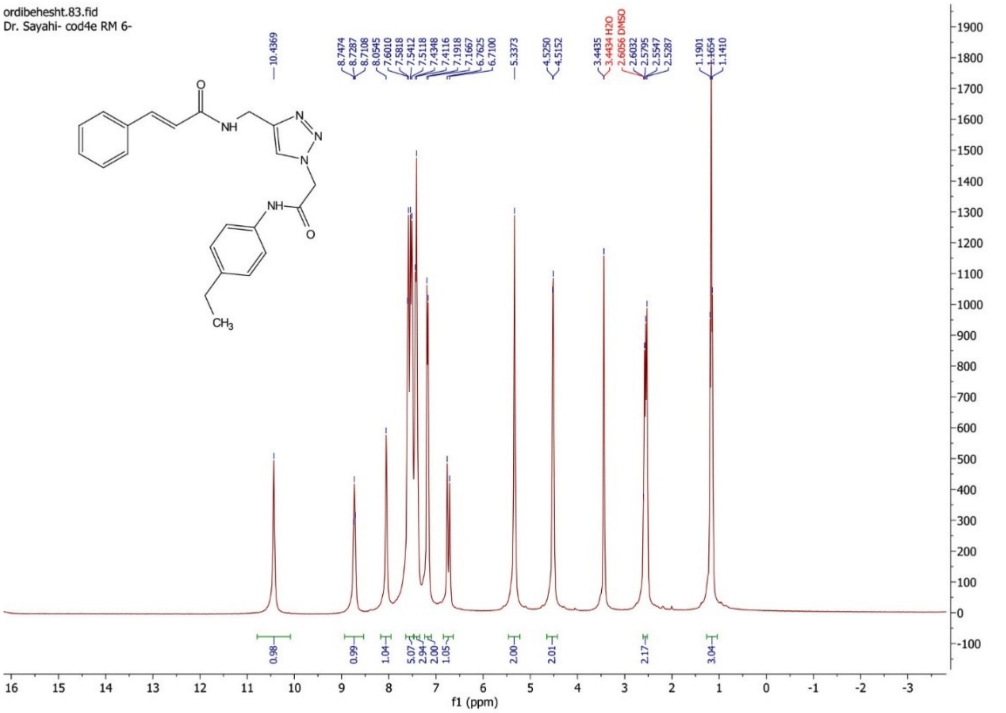

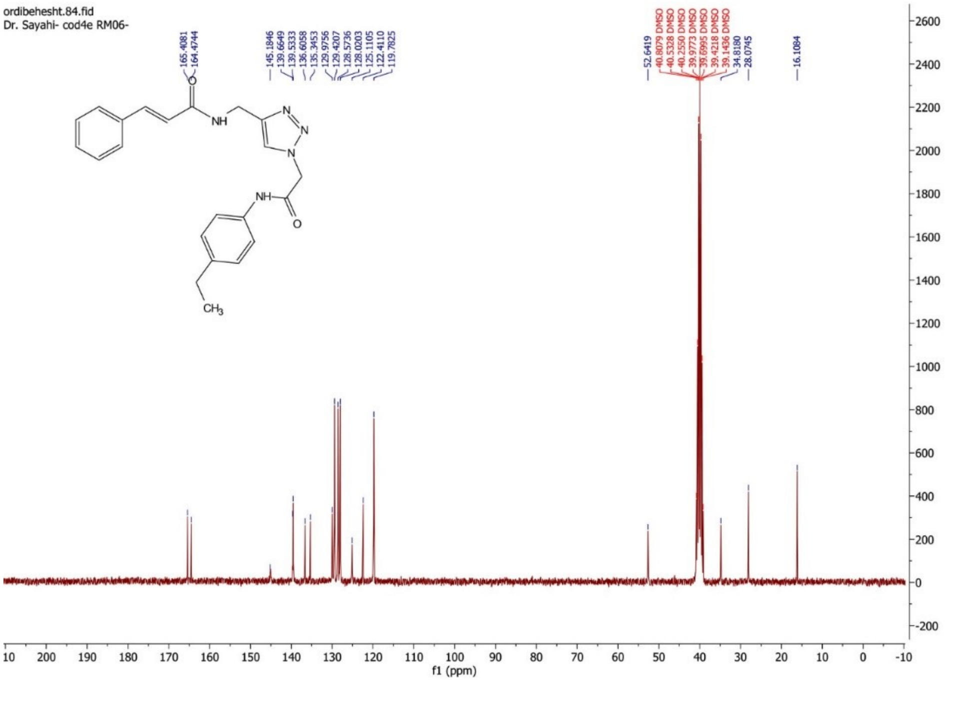

Fig. S6. NMR of 9f

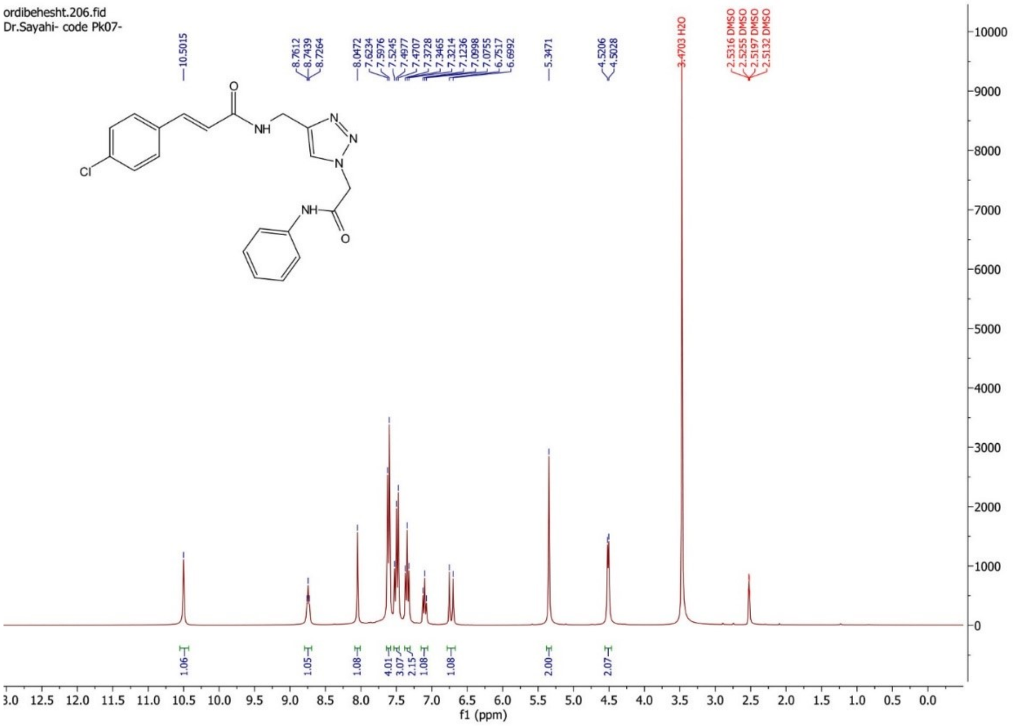

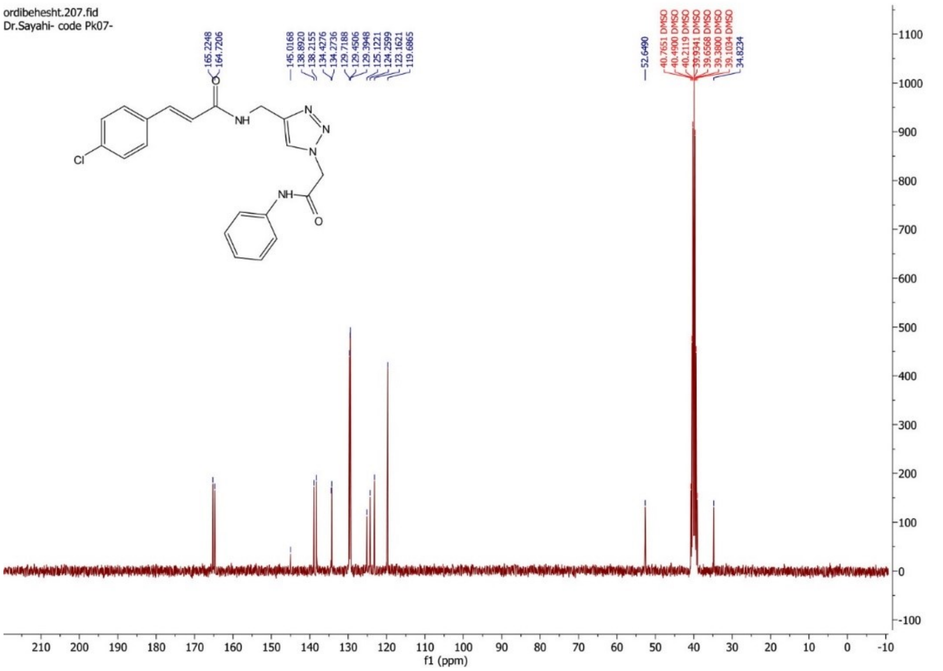

Fig. S7. NMR of 9g

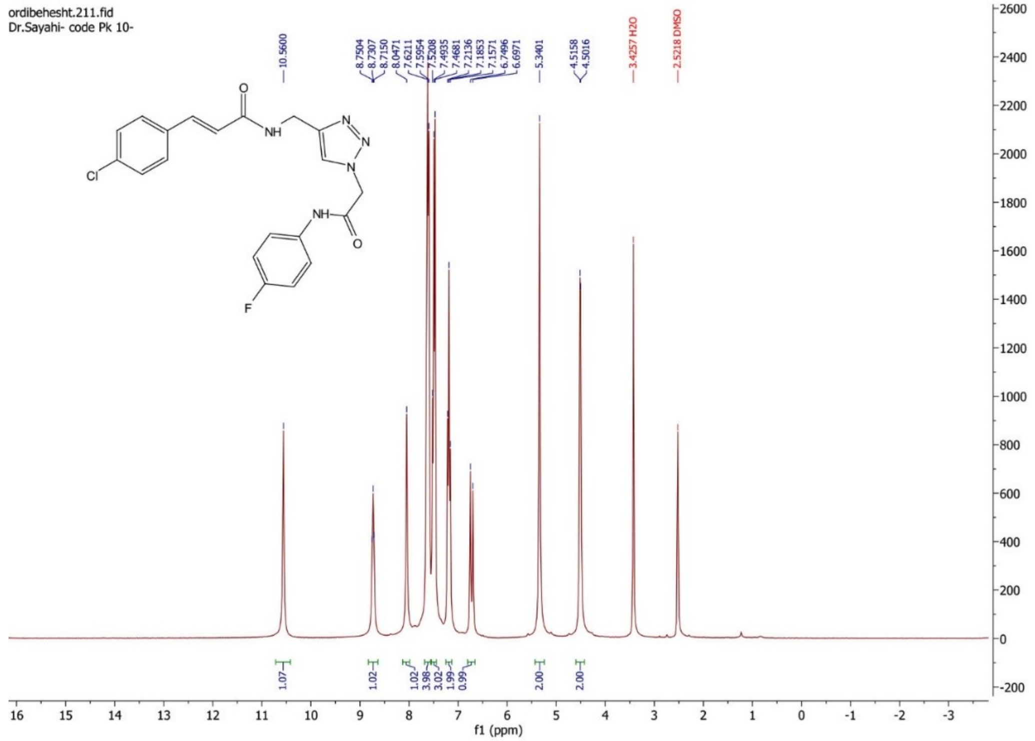

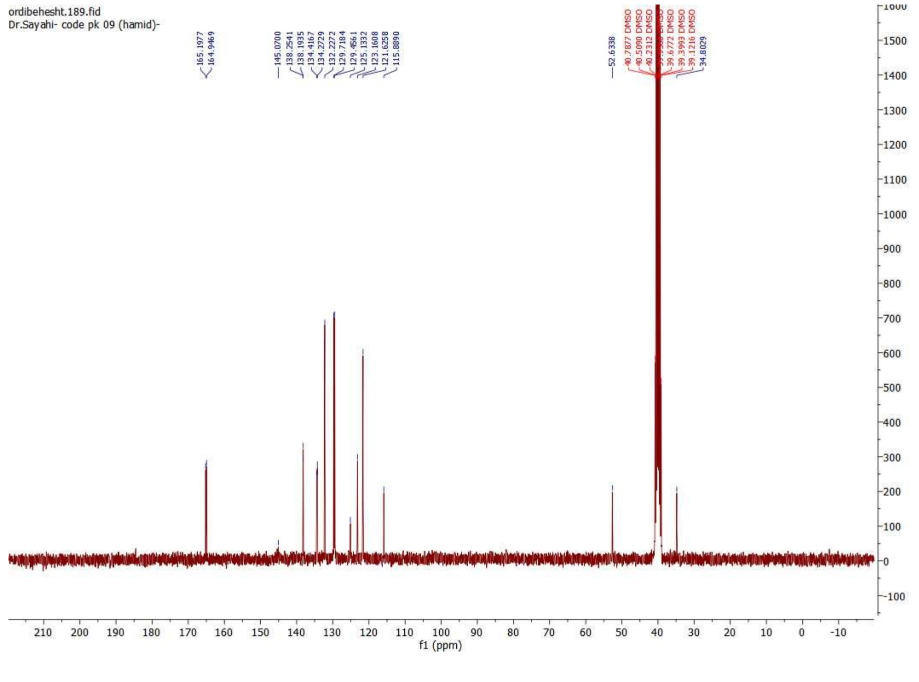

Fig. S8. NMR of 9h

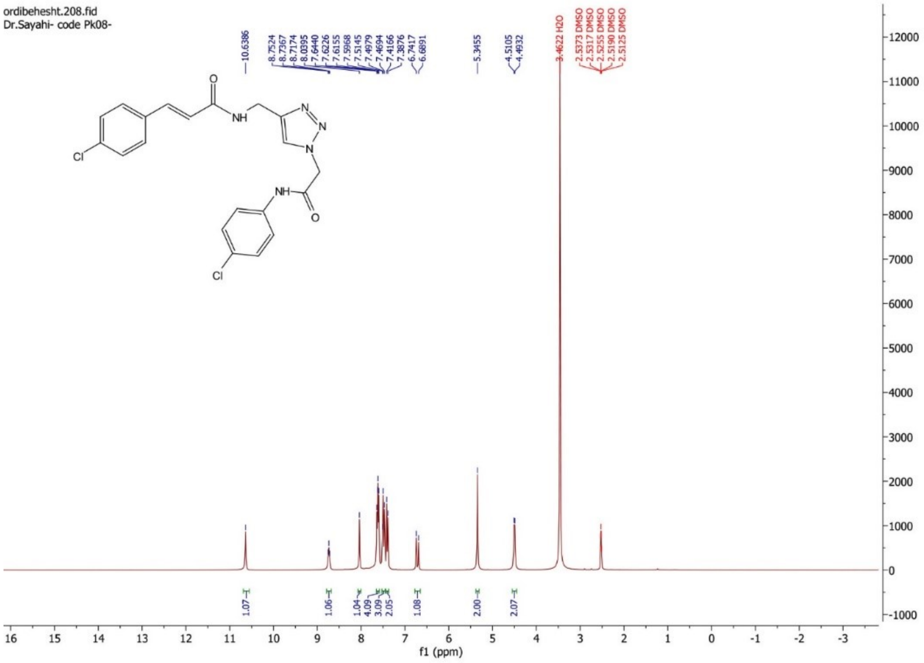

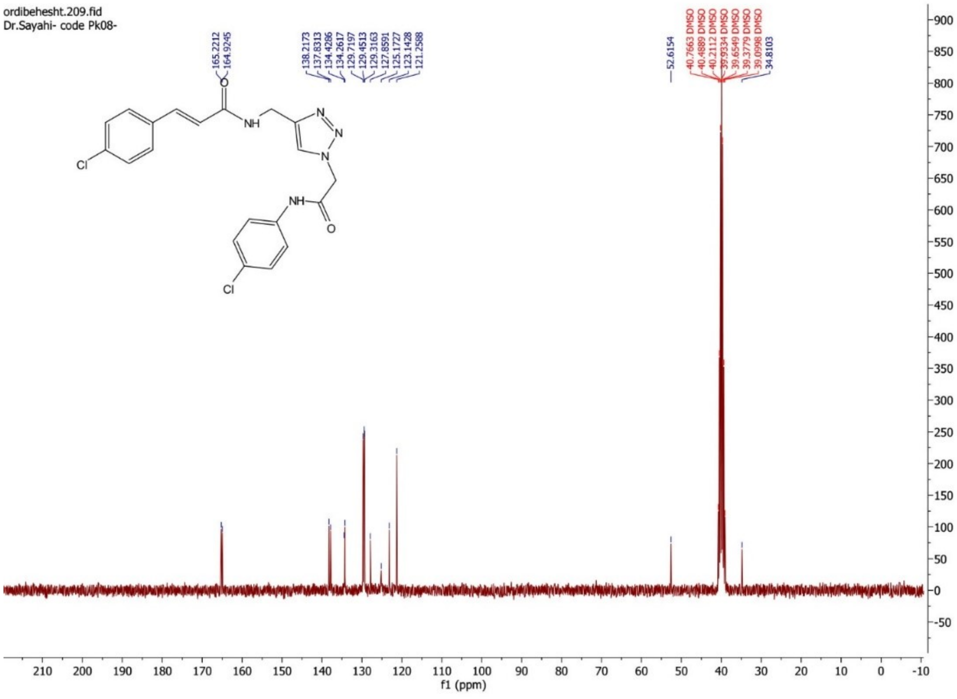

Fig. S9. NMR of 9i

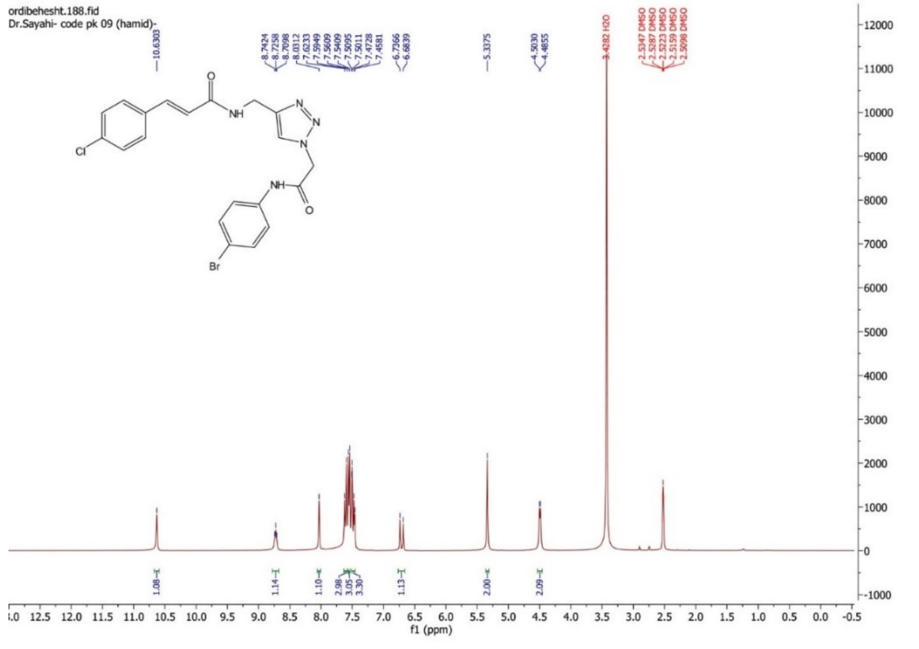

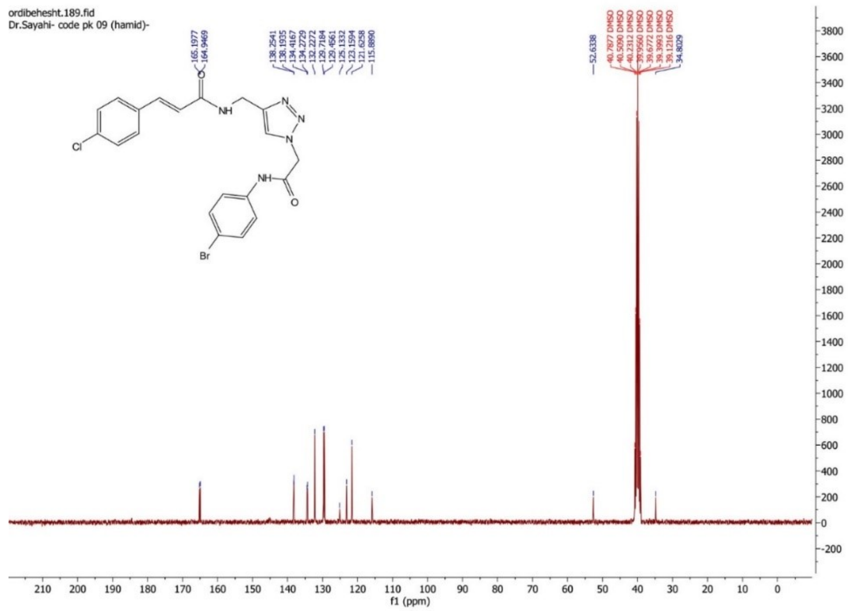

Fig. S10. HPLC of 9i

Empower™ 3 SOFTWARE

SAMPLE INFORMATION

Sample Name:	4-Cl,4Br	Acquired By:	System
Sample Type:	Control	Sample Set Name:	4Cl,4Br
Vial:	6	Acq. Method Set:	
Injection #:	1	Processing Method:	4Cl
Injection Volume:	10.00 ul	Channel Name:	W2489 ChA
Run Time:	70.0 Minutes	Proc. Chnl. Descr.:	W2489 ChA 205nm
Date Acquired:	8/19/2025 10:54:10 PM GST		
Date Processed:	9/6/2025 3:54:26 PM GST		

Auto-Scaled Chromatogram

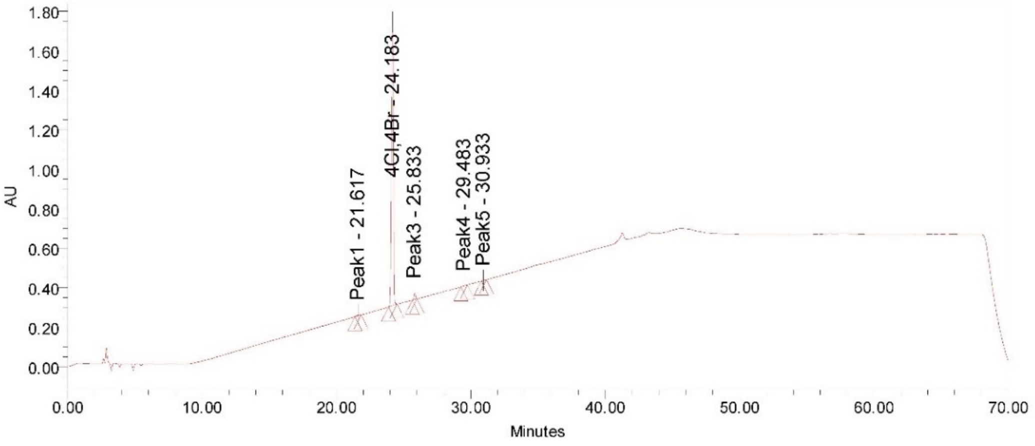

Peak Results

	Name	RT	Area	Height	% Area
1	Peak1	21.617	50030	3068	0.35
2	4Cl,4Br	24.183	14005441	1436464	97.42
3	Peak3	25.833	279388	33563	1.94
4	Peak4	29.483	26118	1747	0.18
5	Peak5	30.933	14907	1445	0.10

Fig. S11. NMR of 9j

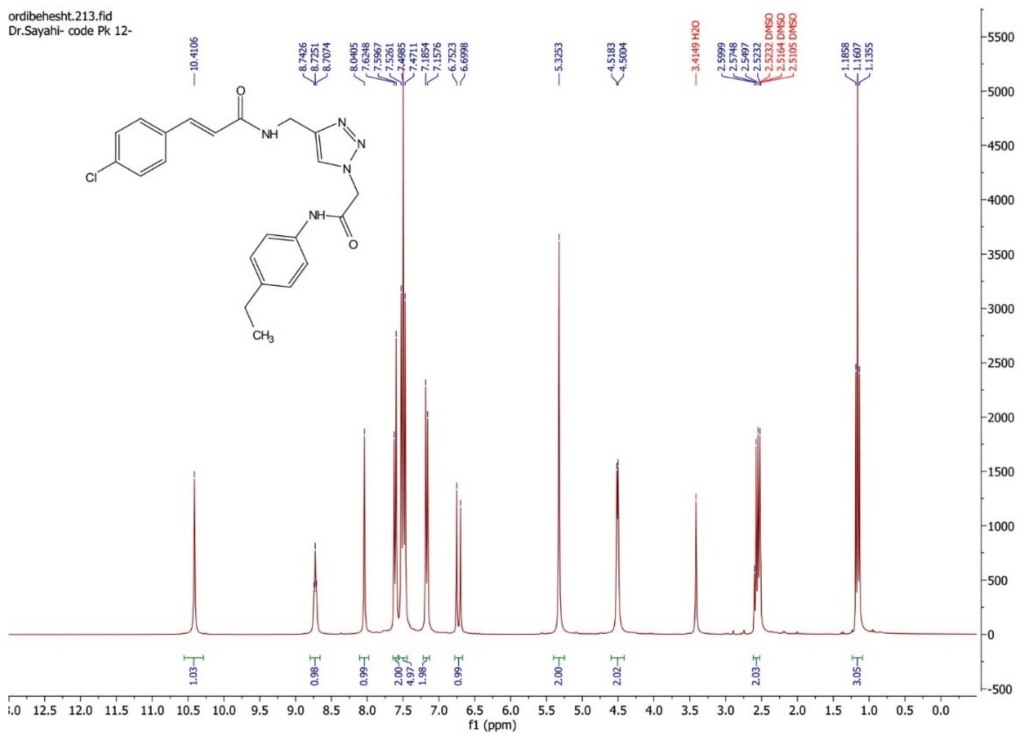

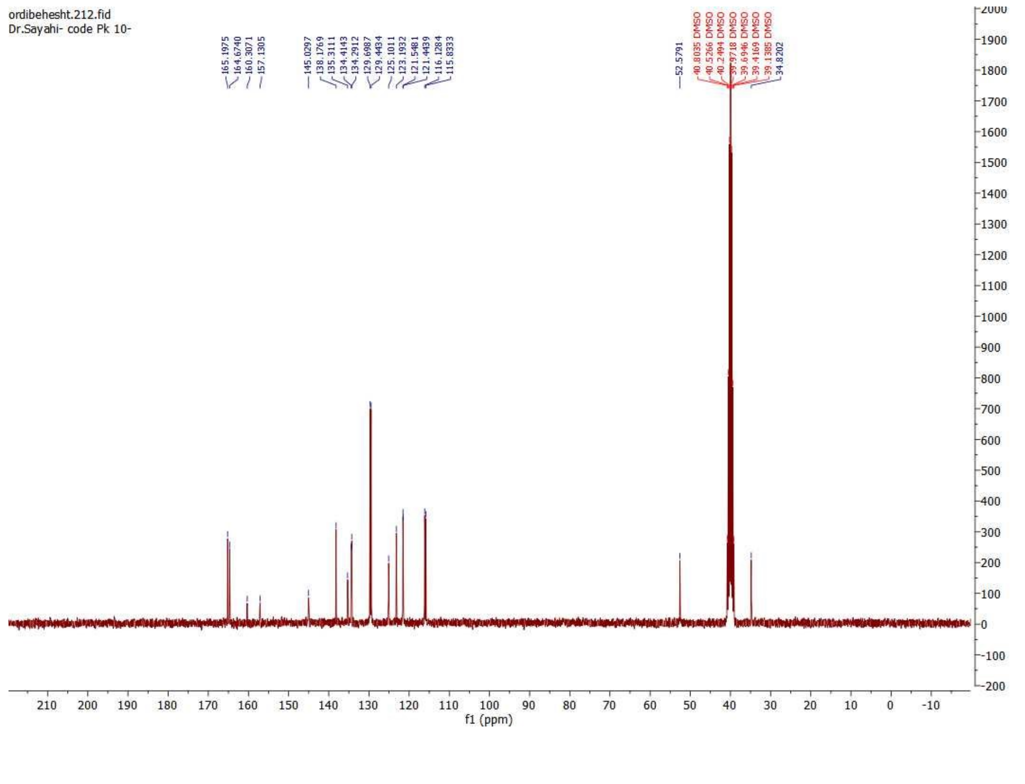

Fig. S12. NMR of 9k

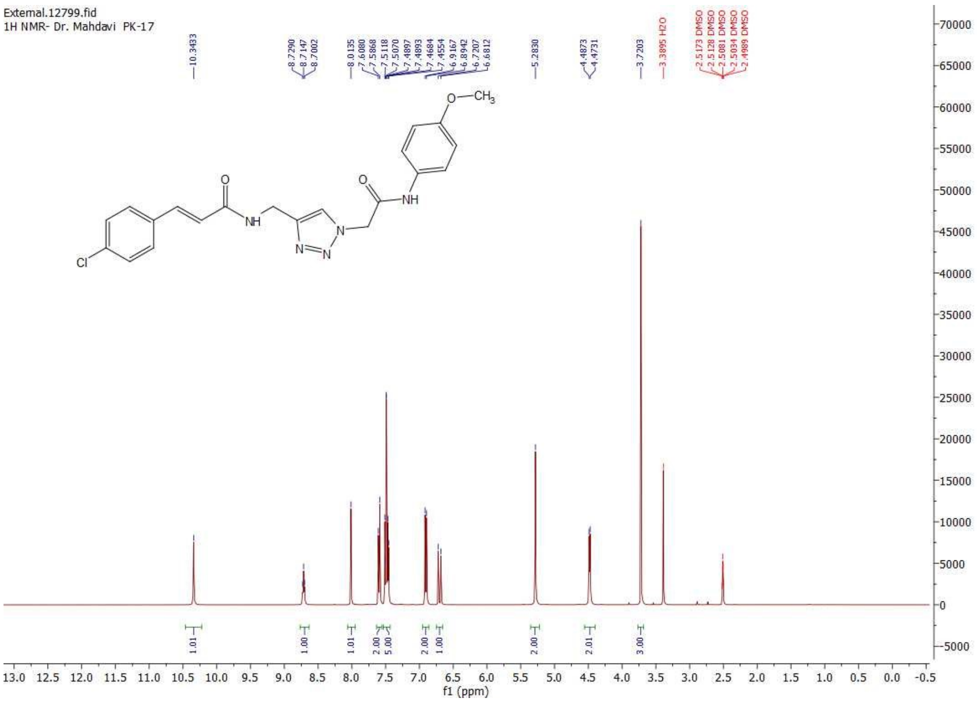

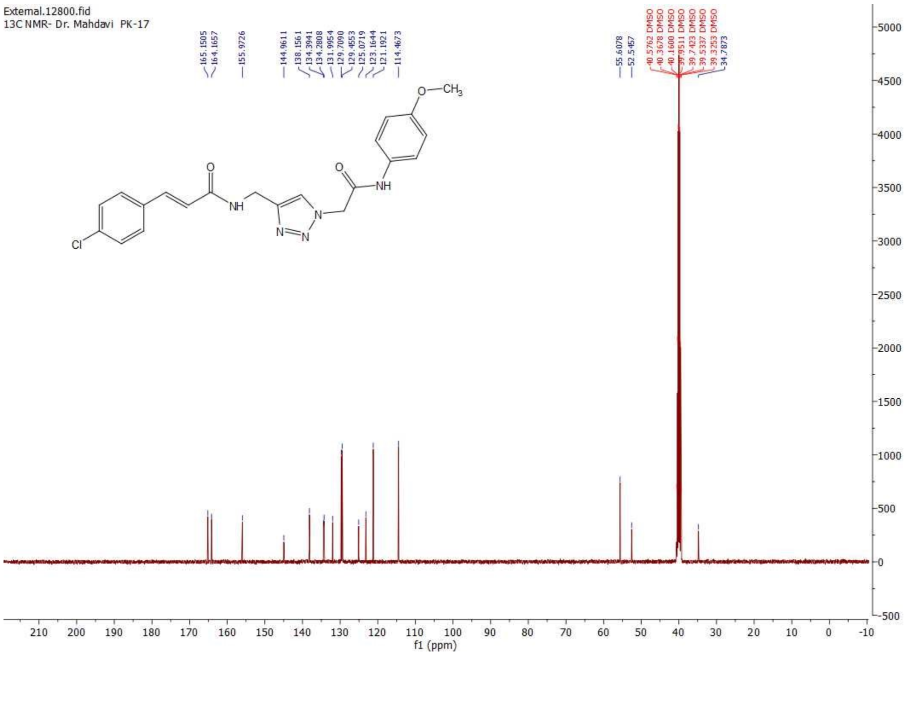

Fig. S13. NMR of 9l

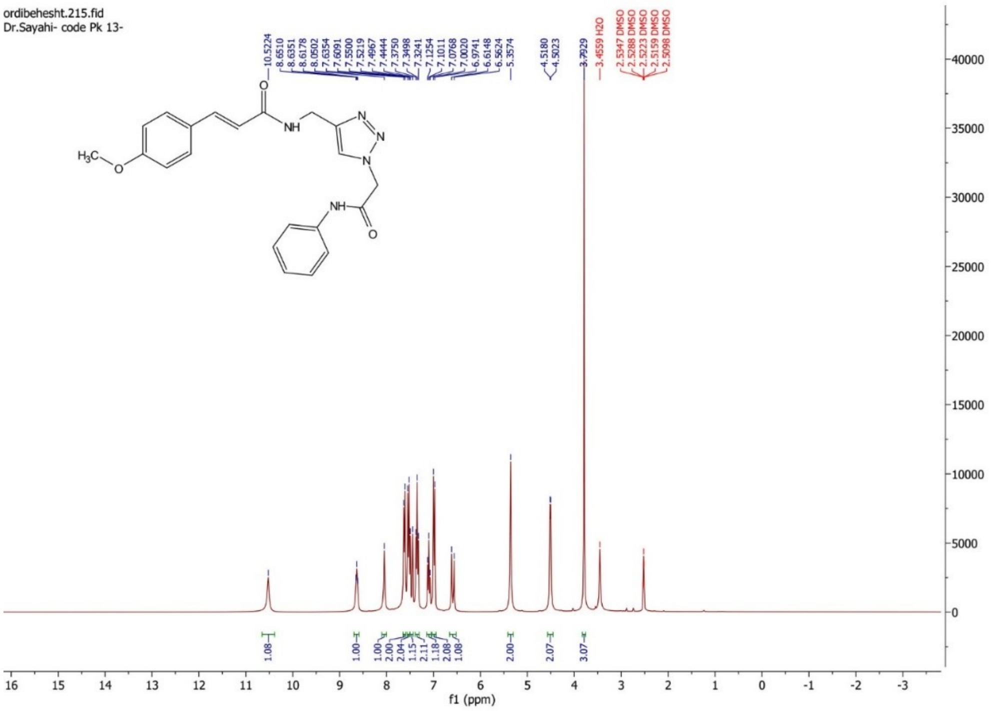

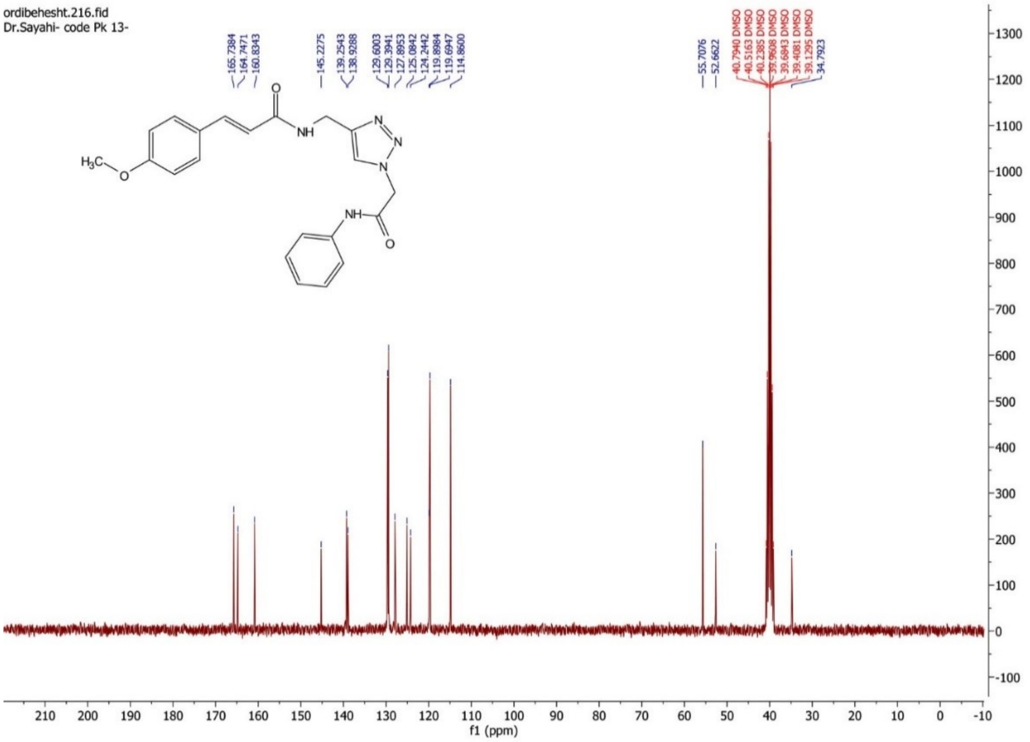

Fig. S14. NMR of 9m

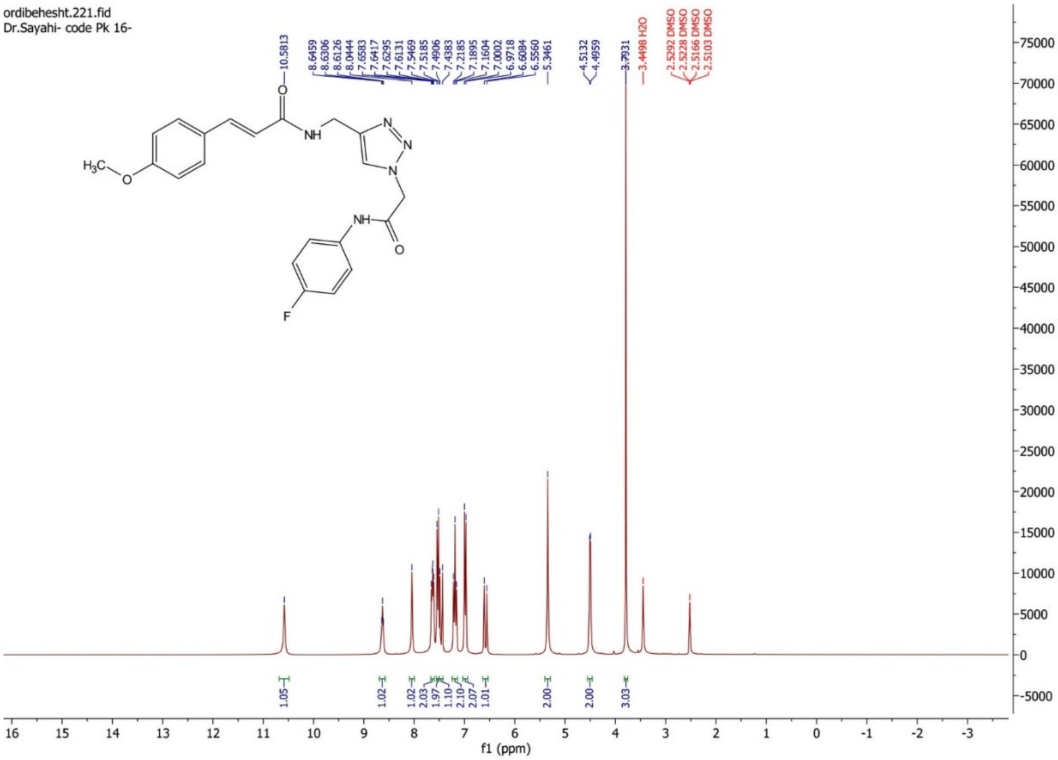

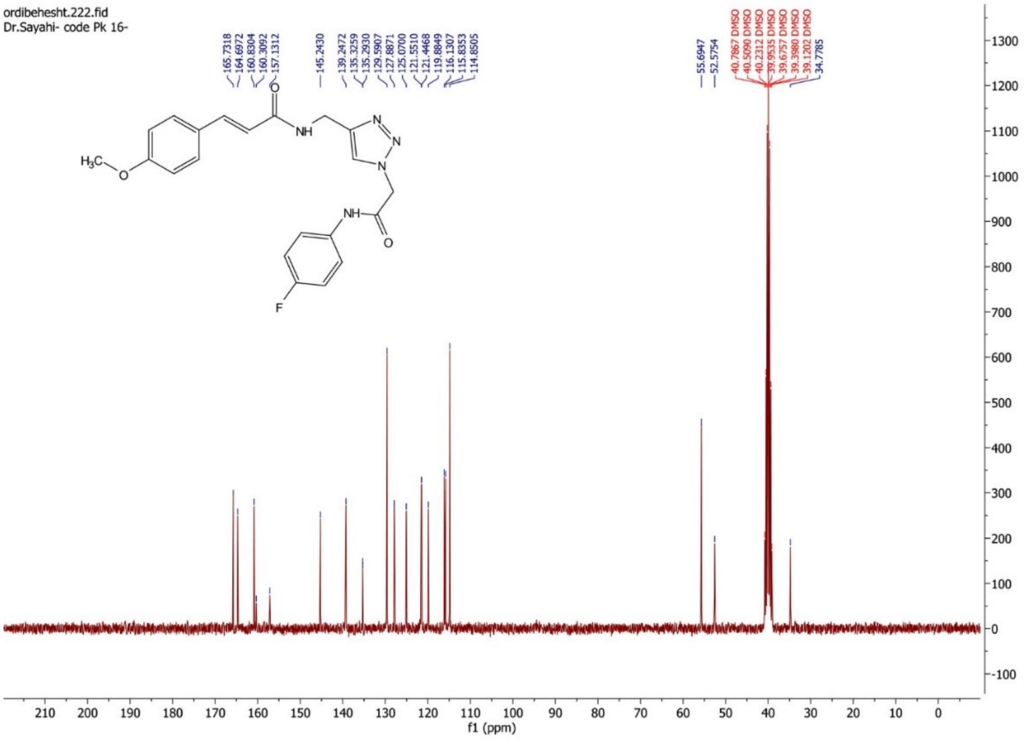

Fig. S15. NMR of 9n

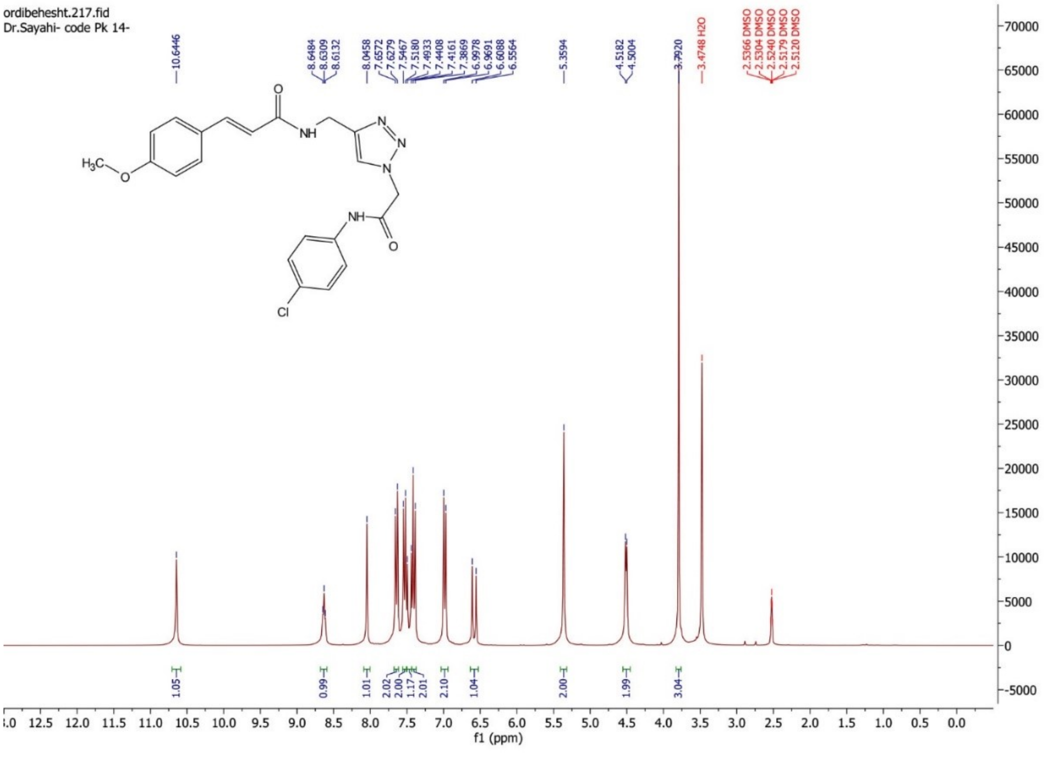

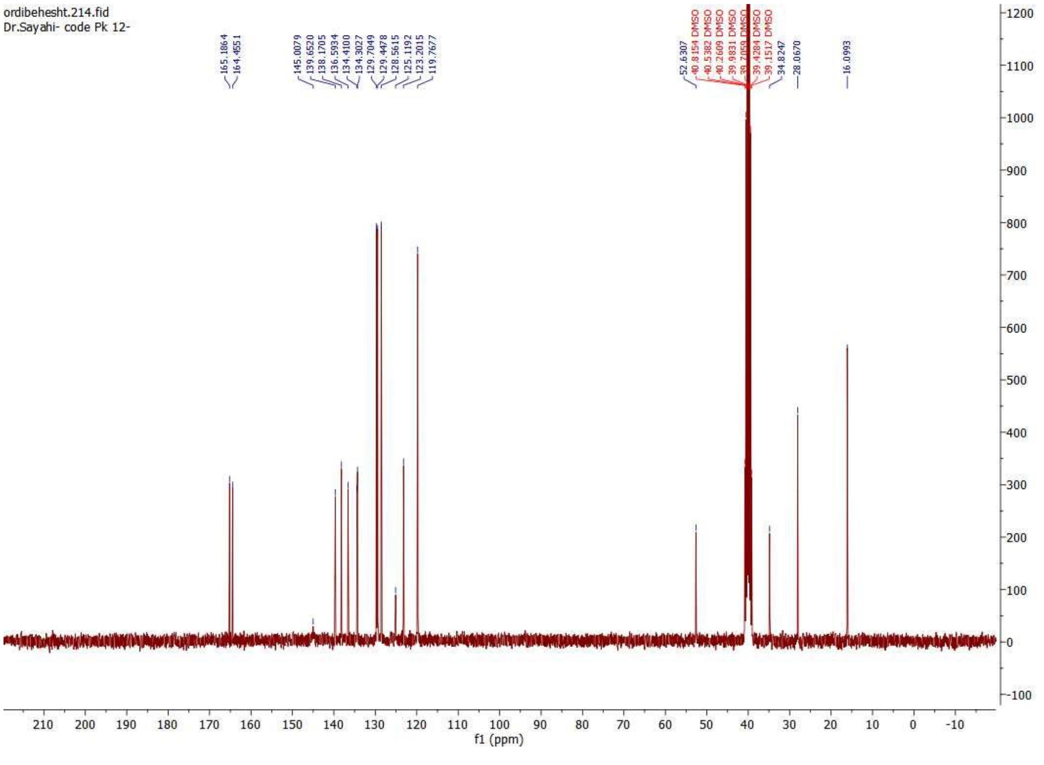

Fig. S16. NMR of 9o

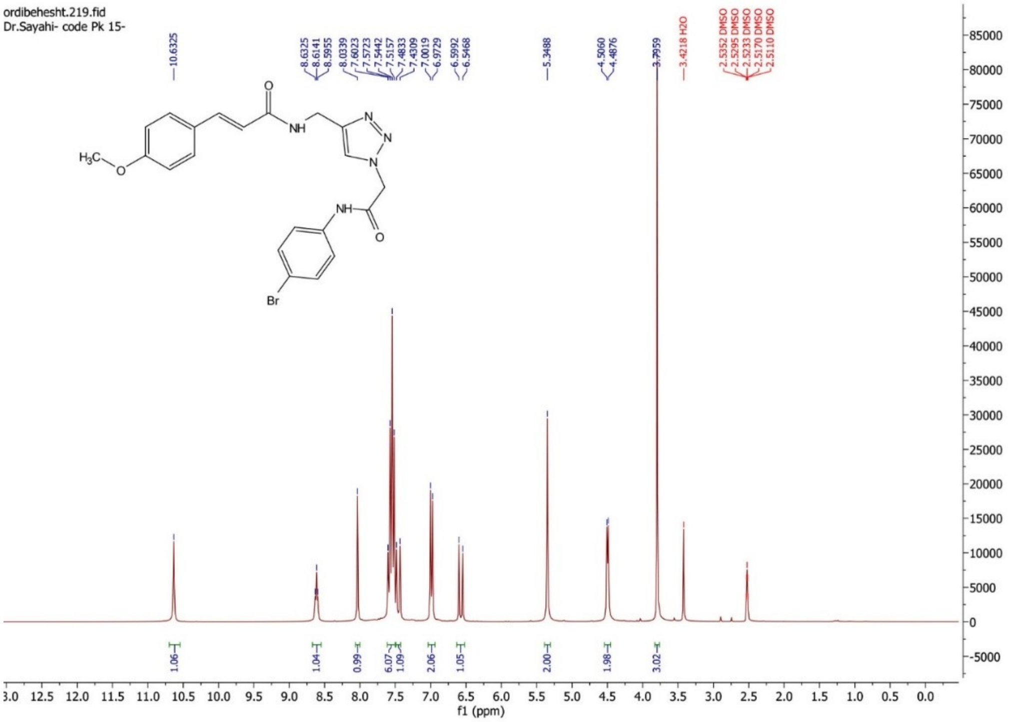

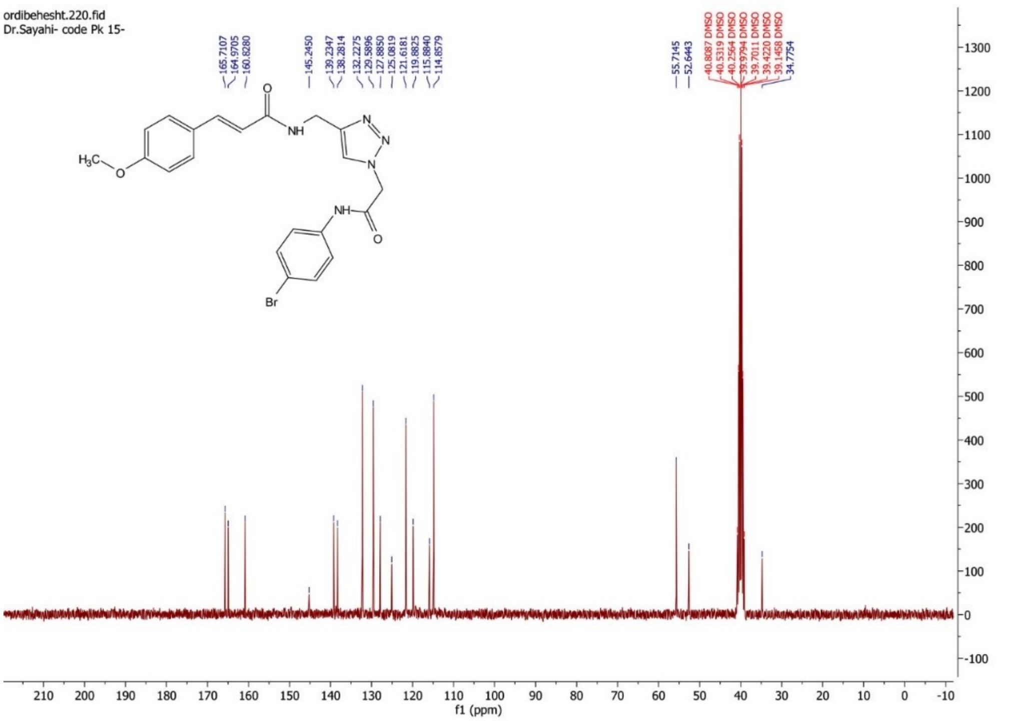

Fig. S17. NMR of 9p

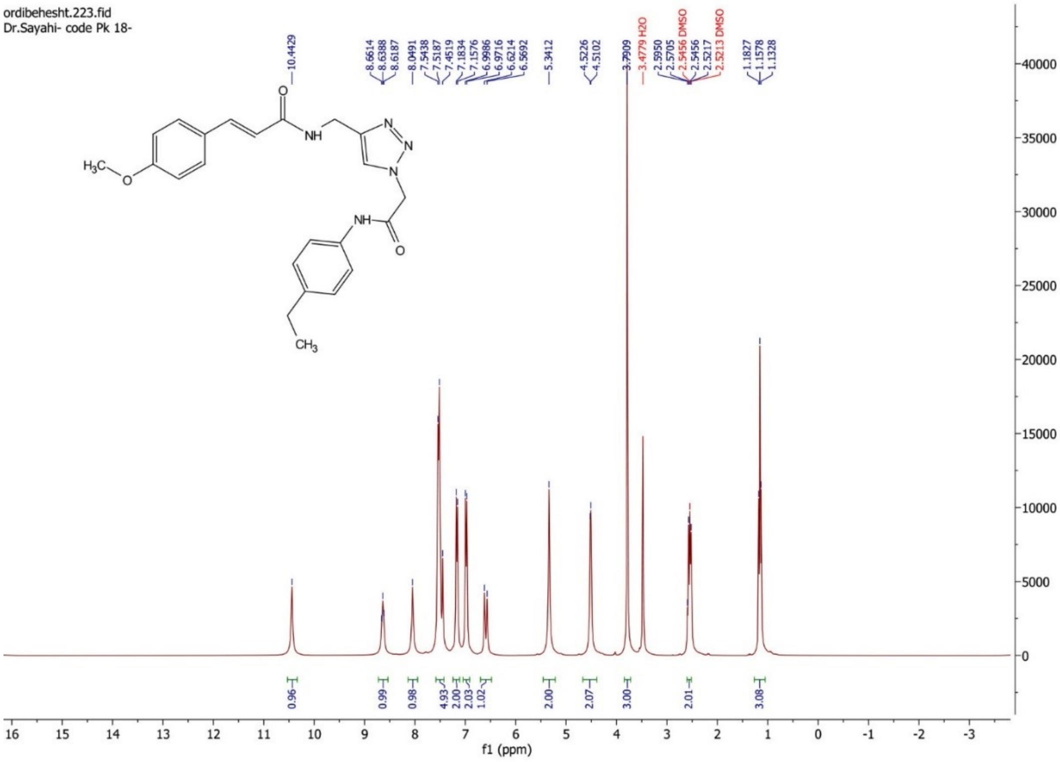

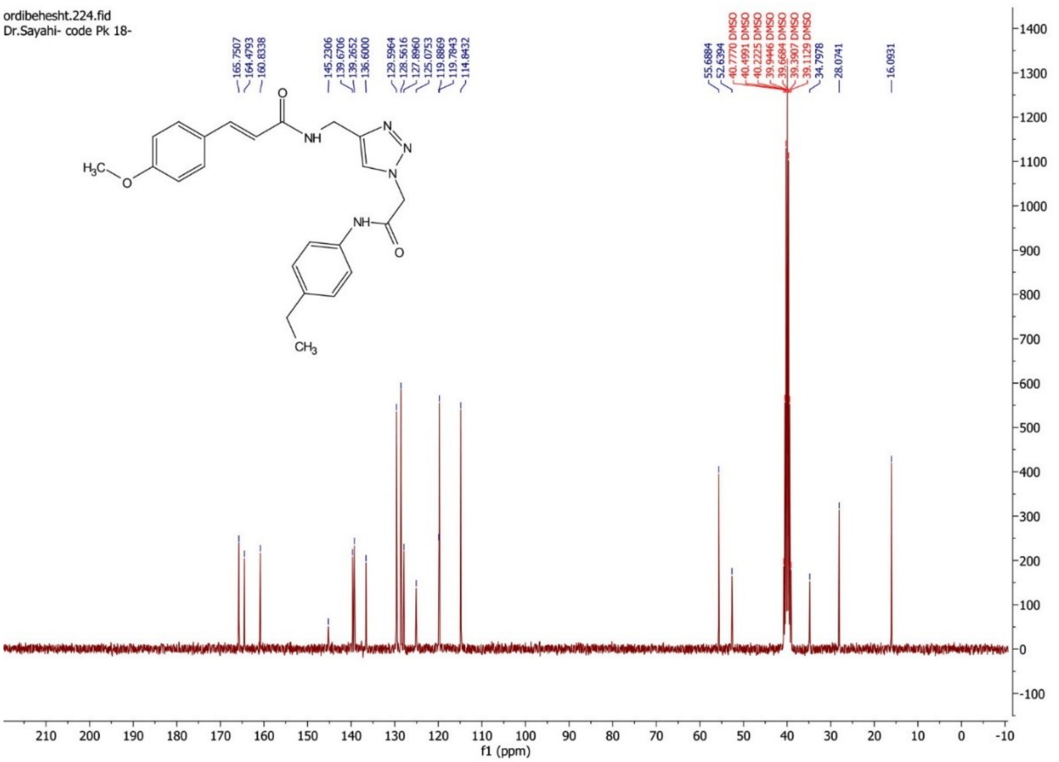

Fig. S18. NMR of 9q

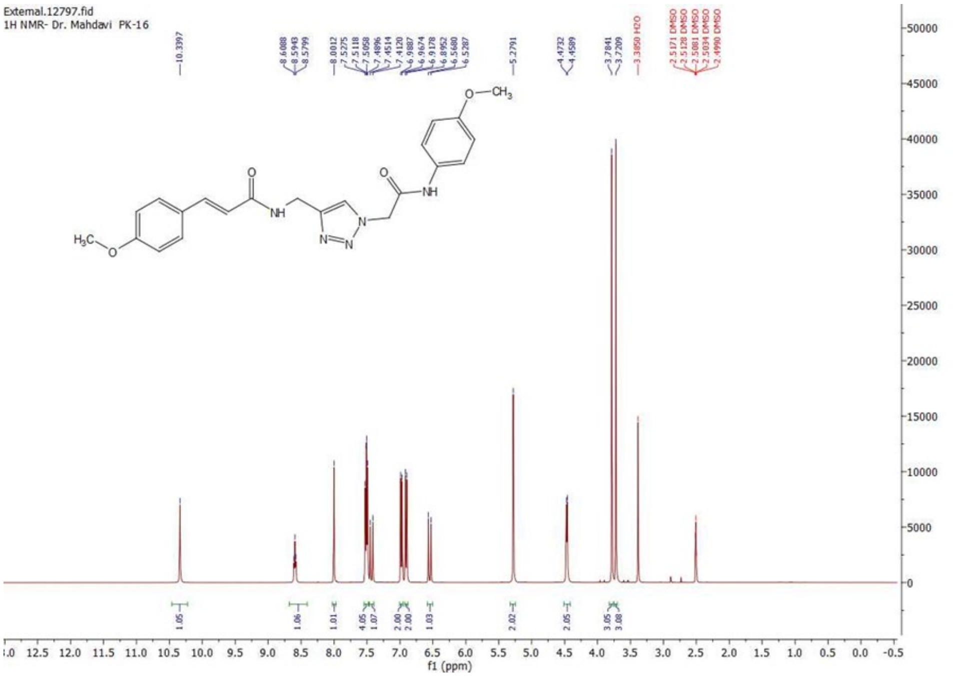

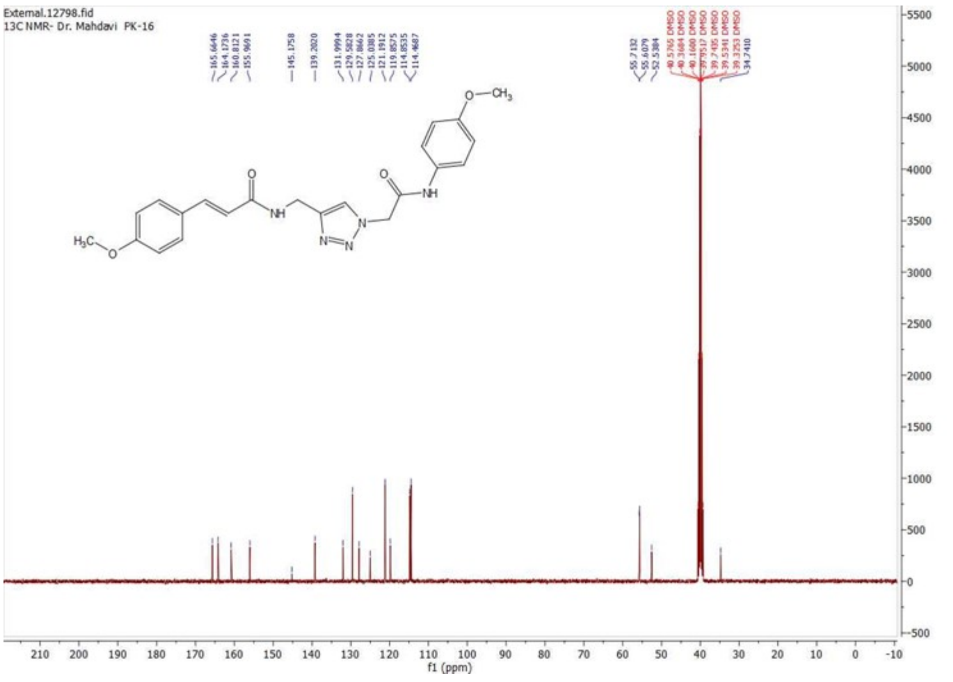

Fig. S19. Mass of 9q

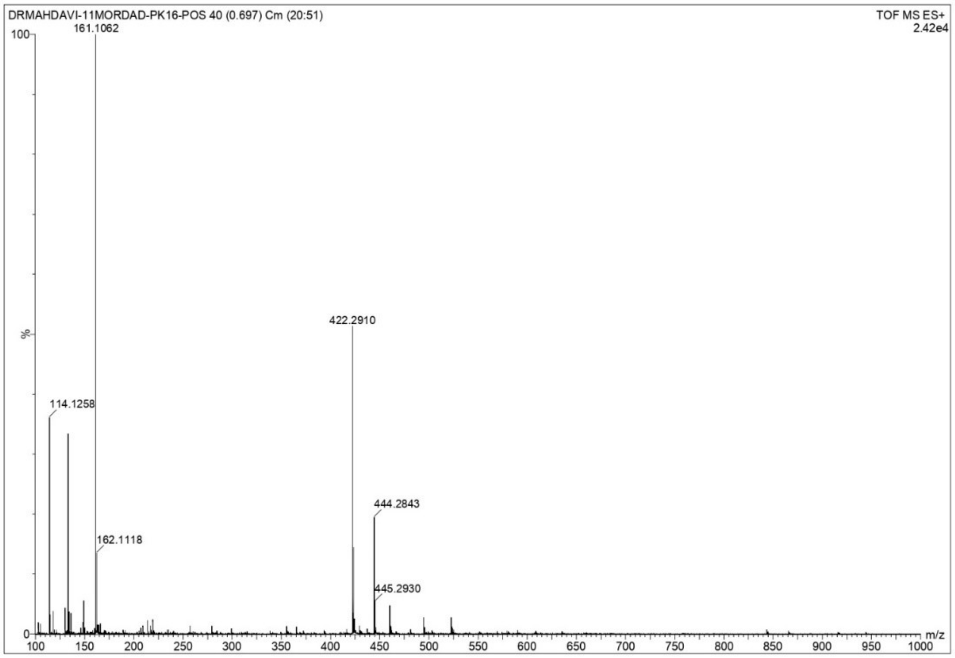

Fig. S20. Ramachandran plot of tyrosinase

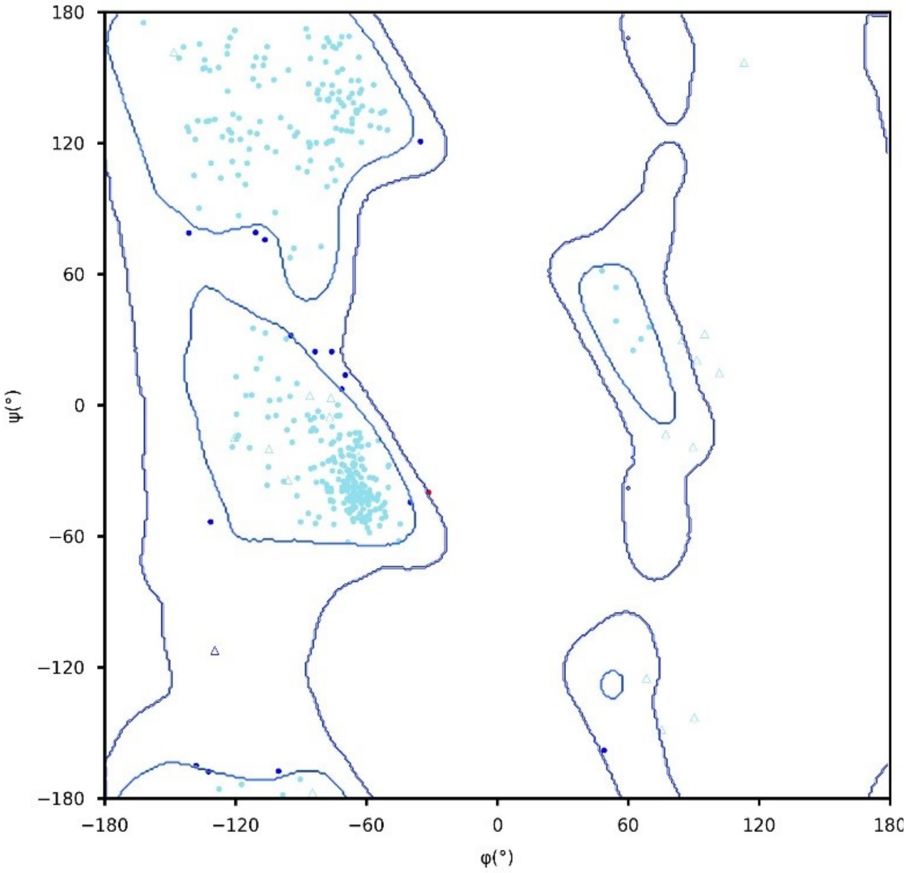

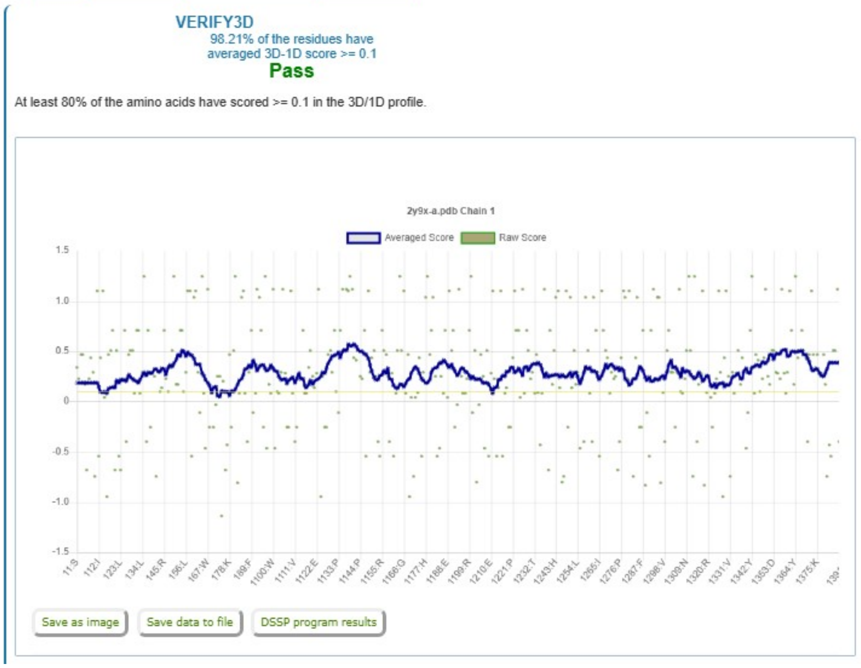

Supplement: RA-015-D5RA04315H-s001 [file RA-015-D5RA04315H-s001.pdf]
